# Targeting CEACAM5-positive solid tumors using NILK-2401, a novel CEACAM5xCD47 κλ bispecific antibody

**DOI:** 10.3389/fimmu.2024.1378813

**Published:** 2024-04-24

**Authors:** Anja Seckinger, Vanessa Buatois, Valéry Moine, Bruno Daubeuf, Françoise Richard, Laurence Chatel, Alizée Viandier, Nicolas Bosson, Emeline Rousset, Krzysztof Masternak, Susana Salgado-Pires, Claudia Batista, Christelle Mougin, Flora Juan-Bégeot, Yves Poitevin, Dirk Hose

**Affiliations:** ^1^ LamKap Bio beta AG, Pfäffikon SZ, Switzerland; ^2^ Department of Hematology and Immunology, Myeloma Center Brussels, Vrije Universiteit Brussel (VUB), Jette, Belgium; ^3^ Light Chain Bioscience (LCB), Plan-les-Ouates, Switzerland

**Keywords:** bispecific antibody, CD47, CEACAM5, immunotherapy, solid cancer

## Abstract

**Background:**

Blocking the CD47 “don’t eat me”-signal on tumor cells with monoclonal antibodies or fusion proteins has shown limited clinical activity in hematologic malignancies and solid tumors thus far. Main side effects are associated with non-tumor targeted binding to CD47 particularly on blood cells.

**Methods:**

We present here the generation and preclinical development of NILK-2401, a CEACAM5×CD47 bispecific antibody (BsAb) composed of a common heavy chain and two different light chains, one kappa and one lambda, determining specificity (so-called κλ body format).

**Results:**

NILK-2401 is a fully human BsAb binding the CEACAM5 N-terminal domain on tumor cells by its lambda light chain arm with an affinity of ≈4 nM and CD47 with its kappa chain arm with an intendedly low affinity of ≈500 nM to enabling tumor-specific blockade of the CD47-SIRPα interaction. For increased activity, NILK-2401 features a functional IgG1 Fc-part. NILK-2401 eliminates CEACAM5-positive tumor cell lines (3/3 colorectal, 2/2 gastric, 2/2 lung) with EC_50_ for antibody-dependent cellular phagocytosis and antibody-dependent cellular cytotoxicity ranging from 0.38 to 25.84 nM and 0.04 to 0.25 nM, respectively. NILK-2401 binds neither CD47-positive/CEACAM5-negative cell lines nor primary epithelial cells. No erythrophagocytosis or platelet activation is observed. Quantification of the pre-existing NILK-2401-reactive T-cell repertoire in the blood of 14 healthy donors with diverse HLA molecules shows a low immunogenic potential. *In vivo*, NILK-2401 significantly delayed tumor growth in a NOD-SCID colon cancer model and a syngeneic mouse model using human CD47/human SIRPα transgenic mice and prolonged survival. In cynomolgus monkeys, single doses of 0.5 and 20 mg/kg were well tolerated; PK linked to anti-CD47 and Fc-binding seemed to be more than dose-proportional for C_max_ and AUC_0-inf_. Data were validated in human FcRn TG32 mice. Combination of a CEACAM5-targeting T-cell engager (NILK-2301) with NILK-2401 can either boost NILK-2301 activity (Emax) up to 2.5-fold or allows reaching equal NILK-2301 activity at >600-fold (LS174T) to >3,000-fold (MKN-45) lower doses.

**Conclusion:**

NILK-2401 combines promising preclinical activity with limited potential side effects due to the tumor-targeted blockade of CD47 and low immunogenicity and is planned to enter clinical testing.

## Background

Different immunotherapeutic approaches exploiting the patient’s immune system to kill tumor cells have shown benefit in a variety of tumor entities, including immunoglobulin G (IgG) monoclonal antibodies (mAbs) targeting antigens expressed on tumor cells (1), approaches targeting the adaptive part of the immune system, for example (re)directing T-cells via chimeric antigen receptor- and T-cell receptor (TCR) modified T-cells, or T-cell bispecific antibodies (BsAb) that simultaneously bind a surface target on tumor cells and an associated TCR chain (CD3 epsilon chain) present on all T-cells (2, 3), as well as the blockade of inhibitory immune checkpoints, e.g., PD-1/PD-L1, CTLA-4, that prevent T-cell responses toward cancer cells (4).

While the focus concerning immune checkpoint inhibition during the last years was largely on the adaptive part of the immune system, recent work has shed light on an innate immune checkpoint, involving the interaction between CD47 on tumor cells and signal regulatory protein alpha (SIRPα) on phagocytes (5). The surface glycoprotein CD47 is an innate immune checkpoint that is ubiquitously expressed on cells of the human body and has been described to be overexpressed on malignant cells from patients with both hematological and solid tumors. It allows tumor cells to escape immune surveillance through its interaction with SIRPα on phagocytes, thus acting as a “don’t eat me signal” (6).

Blockade of CD47 enhances the elimination of CD47-positive tumor cells across multiple preclinical models and has demonstrated efficacy in early phase clinical trials in different tumor entities, including hematological malignancies and solid tumors, while being well-tolerated (7–19).

As CD47 is expressed on all human cells, the immune response needs to be limited to tumor cells as intended target. One strategy to achieve this is the use of BsAbs simultaneously targeting CD47 and a tumor specific antigen (20), here carcinoembryonic antigen-related cell adhesion molecule 5 (CEACAM5, also known as CEA or CD66e), to selectively block CD47 on malignant cells.

CEACAM5, one of the most widely used tumor markers, belongs to the CEACAM family that comprises 12 closely related proteins in humans (21–24). CEACAMs are involved in a variety of physiological processes such as cell-cell recognition and modulate cellular processes ranging from the shaping of tissue architecture and neovascularization to the regulation of insulin homeostasis, and T-cell proliferation; CEACAMs have also been identified as receptors for host-specific viruses and bacteria (25, 26). CEACAM5 is attached to the cell membrane by a glycosyl phosphatidylinositol anchor and released as a soluble form by phospholipase D (27–30). It is present early in embryonic and fetal development and maintains its expression in normal adult tissues. Its main site of expression is in columnar epithelial and goblet cells of the colon, particularly in the upper third of the crypt and at the free luminal surface. Together with the presumably lower expression in healthy tissue, this polarized expression pattern is thought to limit the accessibility to systemically administered therapeutic antibodies (31–34). In contrast, CEACAM5 is (over-) expressed in tumors of epithelial origin, including colorectal, gastric, lung, and pancreatic carcinomas [reviewed e.g., in (23)], where it loses its apical expression resulting in distribution over the entire cell surface (32).

Different molecules targeting CEACAM5 for diagnostic or therapeutic purposes have been tested or are currently in clinical trials, including radiolabeled antibodies, antibody drug conjugates, or T-cell BsAbs both in monotherapy as well as in combination treatment e.g., with checkpoint inhibitors (35–39). Overall, these approaches demonstrated encouraging anti-tumor activity together with a manageable safety profile with adverse events mainly being of grade 1 and 2.

Here we describe the development, preclinical activity, and safety of NILK-2401, a fully human IgG1 BsAb for the treatment of CEACAM5-expressing solid cancers which is based on the so-called κλ body format. This format is assembled by co-expressing one heavy chain and two different light chains, one κ and one λ, resulting in a BsAb that fully retains the sequence and architecture of human antibodies (40).

## Methods

### Cell lines

CEACAM5-positive colorectal, lung, or gastric cancer cell lines were purchased from ATCC (SK-CO-1, SNU-C1, H508, LS174T, SNU-16, H727, H2122) or DSMZ (MKN-45), and cultured according to the respective datasheets. CEACAM5-negative A549 cells (ATCC), and two primary epithelial cell lines, i.e., HBEpiC (ScienCell Research Laboratories) and CCD841CoN (ATCC), were used as controls. The number of CEACAM5 and CD47 antigens/cell was determined by QIFIKIT (Agilent DAKO) according to the manufacturer’s instruction as previously described (41).

MC38 (Kerafast) were silenced for mouse CD47 cell surface expression and were afterwards stably transfected to express human CD47 and human CEACAM5. A stable clone named 3C12 was isolated by cell sorting (Beckman #Cytoflex SRT) and cultured like mentioned in the original MC38 datasheet. The number of human CEACAM5 (203’000 molecules/cell) and human CD47 (40’000 molecules/cell) expressed at the surface of the clone was determined by QIFIKIT (Agilent DAKO) according to the manufacturer’s instruction as previously described (41).

### Antibody-dependent cellular phagocytosis

Peripheral blood mononuclear cells (PBMCs) from healthy donors were isolated from buffy coats (Centre de Transfusion Sanguine Genevois, Switzerland), and monocytes differentiated into macrophages by culturing PBMCs in presence of 20 ng/mL human M-CSF (Peprotech). At least three donors were tested per cell line. To assess the impact of soluble CEACAM5 (sCEACAM5), 0.02, 0.05 and 0.1 μg/mL sCEACAM5 (Biorad) was added to selected experiments.

After six days, differentiated macrophages were detached from the culture flask and 30.000 cells/well seeded in clear flat bottom 96-well plates (Costar). Plates were incubated for 48 hours at 37°C and 5% CO_2_. Subsequently, supernatants form the wells were removed and replaced by 0.5 µM of calcein red-orange (ThermoFisher Scientific) diluted in PBMC medium. After 30 min at 37°C, plates were washed twice with 150 µL of phosphate-buffered saline per well. After the last washing step, 100 µL of PBMC medium containing 2 mg/mL of irrelevant human IgG1 to saturate the FcγR1 expressed on macrophages was added.

In the meantime, target tumor cells were stained with 1 µM of calcein-AM (ThermoFisher Scientific) for 30 min at 37°C. After washing, cells were resuspended at 1.8 x 10^6^ cells/mL and stained target cells were opsonized with antibodies of interest for 20 min at room temperature (RT). Subsequently, 100 µL of opsonized stained target cells were transferred to each well and plates were incubated for 2h 30 min at 37°C. After incubation, medium was removed and replaced with 100 µL of PBMC medium.

Plates were acquired using a CellInsight CX5 imager (ThermoFisher Scientific) and values corresponding to the average number of target cells number engulfed per macrophage were extracted using the imager’s HCS Studio software. Values were used to calculate the phagocytosis index, defined as the number of target cells engulfed by one hundred macrophages.

### Antibody-dependent cellular cytotoxicity

PBMCs from healthy donors were used as a source of effector T-cells. At least three donors were used for each cell line tested. To assess the impact of sCEACAM, 0.02, 0.05 and 0.1 μg/mL sCEACAM5 was added to selected experiments.

PBMCs were centrifuged at 300 g for 5 min and the cell pellet resuspended in Roswell Park Memorial Institute (RPMI) 1640 medium supplemented with 2% fetal calf serum (FCS), 2 mM L-glutamine, 25 µg/mL gentamicin (all from Sigma-Aldrich), at a density of 1x10^7^ cells/mL. 5x10^5^ PBMCs were plated in Ultra-Low Attachment Sterile 96-well round bottom plates (Corning) and 1x10^4^ target cells added. Antibodies were diluted in RPMI-1640 containing 2% (v/v) FCS at 4x final concentrations and 25 µL of the diluted antibody was added to the plate. The highest final concentration was 6.66 nM, followed by seven 1/5 serial dilutions. Wells containing only effector cells mixed with target cells, or target cells only, served as controls. Plates were incubated at 37°C for six hours.

For readout, 5 µL of lysis buffer (Cytotoxicity detection kit PLUS (LDH), Roche) was added to wells containing only the targets cells and incubated for at least 5 min. The lysis step was verified by visual inspection under the microscope. These wells served as positive control of 100% target cell lysis.

In the meantime, the detection mixture was prepared according to the manufacturer’s instructions. 100 μL of the freshly prepared detection mixture was added to the plate containing the diluted ADCC samples and incubated at RT, protected from light, for 15 min. After incubation, 50 μL of stop reagent was added. Optical density was measured using a microplate reader at 490 nm (Spectra i3Max). The percentage of specific lysis was calculated by using the following equation:


Specific lysis%=(sample value−(effector+target))/(max. target−target)×100%

Effector + target=baseline without Ab; max. target=target cells with lysis buffer; target=target cells only (spontaneous LDH release).

Specific lysis results were then reported in GraphPad Prism to establish potency curves.

### *In vivo* models

Animal studies were performed in accordance with the Swiss Veterinary Office guidelines and approved by the Cantonal Veterinary Office (Geneva, Switzerland; #GE134 and #GE299). Mice were randomly assigned into different groups. Study endpoints included tumor volume >1500 mm^3^, body weight loss >15%, and other graft-versus-host-disease symptoms. Anesthesia was performed by inhalation of isoflurane 3%, for 10 min. maximum, euthanasia with an intraperitoneal (IP) injection of pentobarbital 150 mg/kg (lethal dose).

In the xenograft model, 7-8-week-old NOD SCID female mice (NOD.Cg-Prkdc^scid^/J; Charles River) were injected subcutaneously (SC) with 3x10^6^ SNU-C1 target cells. Treatment with NILK-2401 intravenously (IV) at 20 mg/kg or IgG1 isotype control at 20 mg/kg (n=8 each) twice/week was started on day 1. The last injection on day 43 was given IP for technical reasons.

NILK-2401 was also tested in a syngeneic model with 6- to 20-week-old female human CD47/human SIRPα transgenic mice (C57BL/6-Sirpa^tm1(SIRPA)^Cd47^tm1(CD47)^/Bcgen; Biocytogen) engrafted SC with 0.5x10^6^ MC38-hCEA-hCD47 cells expressing human CEACAM5 and human CD47 (**Supplementary Table S1**). Treatment was started at day 1 post engraftment; NILK-2401 was dosed IP at 30 mg/kg twice/week (n=6), an IgG1 isotype antibody at 30 mg/kg served as control (n=8).

Mice were controlled daily for clinical symptoms and potential adverse events. Animals were weighed twice/week using a digital balance. For tumor growth follow-up, tumors were measured thrice/week in two dimensions (length, width) using a caliper and the volume was expressed in mm^3^ using the formula:


$$Tumor\;volume\;=\;\left(length\;\times \;width^{2}\right)\;\times \;0.5.$$

The tumor volume measurement was used to calculate the area under the curve (AUC) for each individual mouse. To estimate the AUC, the same time frame was used for all mice. The statistical analysis of AUC was performed with data starting from the day of the engraftment (d0), until the first day of euthanasia due to tumor volume >1500 mm^3^. AUC reflects the tumor growth curve between two defined time points and allows comparison between groups.

The percentage of tumor growth inhibition (TGI) at indicated time points in comparison to the control group was determined based on tumor volumes, using the formula:


%TGI=1−[(Tt–T0)/(Vt−V0)] × 100%.

Tt = mean tumor volume of treated at time t. T0 = mean tumor volume of treated at time 0. Vt = mean tumor volume of control at time t. V0 = mean tumor volume of control at time 0.

A Kaplan-Meier survival analysis was done using GraphPad Prism software. Mice euthanized due to ulceration were excluded from the analysis.

Please see the **Supplementary Data** for additional information.

## Results

### Development of NILK-2401

**Figure 1:**
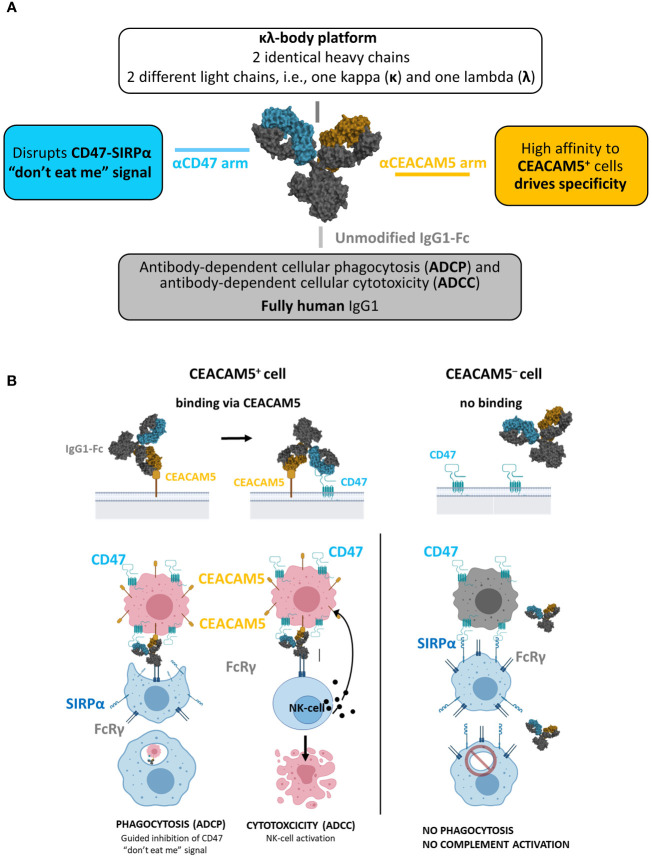
NILK-2401. **(A)** Schematic depiction of the bispecific antibody, aiming at blocking the CD47 “don’t eat me” signal which allows tumor cells to escape immune surveillance through its interaction with signal regulatory protein alpha (SIRPα) on phagocytes. **(B)** Envisioned mechanism of action: 1) Initial binding of NILK-2401 to CEACAM5^+^ tumor cells driven by the high affinity anti-CEACAM5 arm. 2) Co-engagement and blockade of CD47 on tumor cells driven by the low affinity anti-CD47 arm. 3) Fc-mediated effector cell recruitment, fully functional IgG1 Fc. 4) Tumor cell killing via antibody-dependent cellular phagocytosis (ADCP) and antibody-dependent cellular cytotoxicity (ADCC).

The κλ body platform (40) was used to generate a human CEACAM5xCD47 BsAb selectively blocking CD47 on CEACAM5-positive tumor cells (**Figure 1**).

The anti-CD47 arm was isolated from the naïve dummy kappa libraries using LCB’s phage display platform. Due to ubiquitous CD47 expression, the anti-CD47 arm was designed to minimize CD47-mediated monovalent binding of the CEACAM5xCD47 BsAb to CEACAM5-negative cells. The selection of the final anti-CD47 arm (called K2) was based on *in vitro* and *in vivo* pharmacology, pharmacokinetic and toxicology/tolerability studies to identify the CD47-arm with the most appropriate affinity to maximize efficacy on one hand and tolerability on the other hand. A detailed description of the characterization of the anti-CD47 arm is summarized in (42).

The isolation of anti-CEACAM5 sequences was performed using proprietary phage display libraries displaying scFv from the dummy naïve lambda libraries. Fifty-eight different selection strategies were performed using soluble recombinant human CEACAM5 and/or cynomolgus CEACAM5, or cells expressing human CEACAM5 or cynomolgus CEACAM5, as well as combinations of both approaches. Generated κλ bodies were tested in several *in vitro* assays, to assess their activity and safety, and the most promising candidates were subjected to an affinity maturation process (lead optimization). Following the in-depth characterization of lead-optimized candidates, a candidate matching the target product profile was identified (i.e., K2AC100), selected as clinical lead candidate, and termed NILK-2401.

### Characterization of NILK-2401

#### Target affinity and cross-reactivity

Binding affinity of NILK-2401 on human CEACAM5 protein was in the same nanomolar range for both NILK-2401 batches tested and ranged from 3.9 to 5.3 ± 0.7 nM, independently of the recombinant protein used. NILK-2401 does not show cross-reactivity with cynomolgus CEACAM5. For the “K2” anti-CD47 arm, an affinity of 454 ± 44 nM for human and 526 ± 61 nM for cynomolgus CD47 was measured. The higher binding affinity to CEACAM5 as compared to CD47 enables the selective blockade of CEACAM5-expressing tumor cells.

To determine the CEACAM5-binding region and key residues, epitope binning was performed by assessing the binding of NILK-2401 to recombinant CEACAM5 protein in the presence of several reference antibodies, targeting different domains on CEACAM5. The anti-CEACAM5 arm of NILK-2401 was found to compete in a dose-dependent manner with reference antibody SM3E, which is known to bind to the N-terminal domain of CEACAM5. To determine the binding epitope of NILK-2401, two mutagenesis strategies were applied, identifying two residues as essential for NILK-2401-binding to human CEACAM5: S66 and H123.

#### Selectivity

NILK-2401 binds selectively to CEACAM5; only weak binding (K_D_≈700 nM) to CEACAM3, a receptor expressed in neutrophils, was observed (**Supplementary Figure S1**). No binding to the other members of the family was detected.

### *In vitro* activity and mechanism of action

#### Cell binding

**Figure 2:**
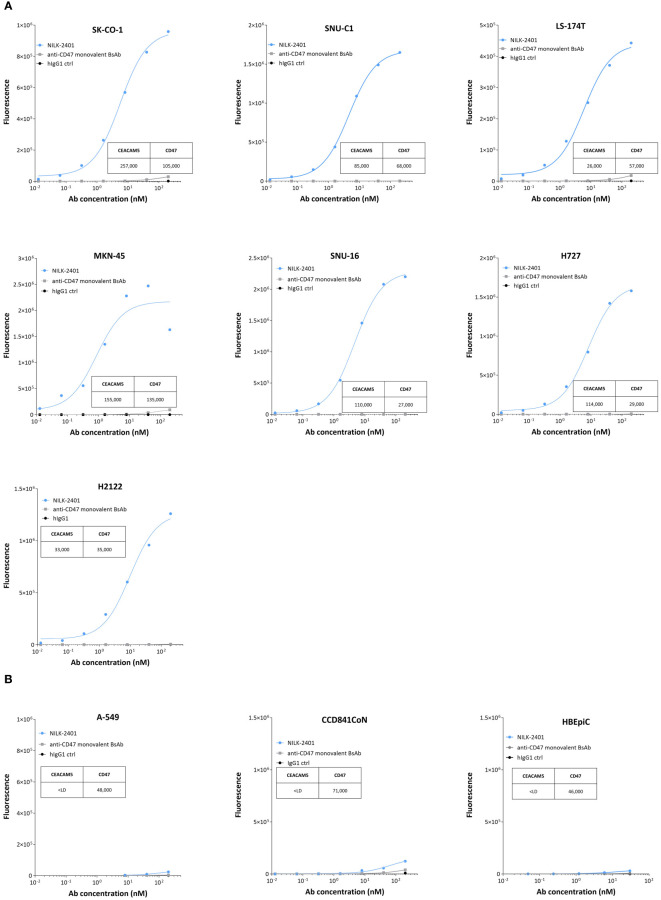
Binding of NILK-2401 to CEACAM5-positive and -negative cell lines as well as primary epithelial cells. Dose-dependent binding profile of NILK-2401 on **(A)** CEACAM5-expressing cancer cell lines SK-CO-1, SNU-C1, LS174T, MKN-45, SNU-16, H727, and H2122. **(B)** Absence of binding was observed for CEACAM5-negative cell line, A549 and to CCD841CoN, isolated from normal colon, as well as HBEpiC, isolated from normal bronchial tissue. BsAb, bispecific antibody; ctrl, control. Graph is representative of two independent experiments. Acquisition was performed on at least 10,000 cells, one simplicate per condition.

*In vitro*, NILK-2401 binds dose-dependently to colorectal (3/3), gastric (2/2) and lung carcinoma (2/2) cell lines with different CEACAM5 expression levels (**Figure 2**, **Supplementary Table S1**). As expected, no binding was observed to CEACAM5-negative cells (A549) or normal epithelial cells, i.e., CCD841CoN and HBEpiC. The monovalent BsAb variant, which carries the same CD47-arm as NILK-2401 but paired with a non-binding arm, showed a very weak binding to the CEACAM5- and CD47-positive cell lines, confirming that NILK-2401 binds to CEACAM5-positive cell lines mainly through its CEACAM5-binding arm. We next performed a binding assay in the presence of 0.02, 0.05 and 0.1 µg/mL of sCEACAM5 with the tumor cell lines SK-CO-1, MKN-45, and H2122. As shown in **Supplementary Figure S2A**, sCEACAM5 had little impact on the binding of NILK-2401 to the CEACAM5-positive cell lines tested (e.g., 30% shift in the AUC with the highest concentration of sCEACAM5 for MKN-45).

#### CD47/SIRPα inhibition

Results of the CD47/SIRPα inhibition assay demonstrated that NILK-2401 inhibited the interaction of the human SIRPα-Fc with CD47 expressed on the cell surface of MKN-45 target cells, in a dose-dependent manner, with a higher blocking activity than the corresponding CD47 monovalent control, indicating that efficient CD47 inhibition is dependent on CEACAM5 co-engagement (**Supplementary Figure S3**).

#### Receptor occupancy

Based on the binding profiles, the percentage of receptor occupancy (RO) at different NILK-2401 concentrations was calculated. NILK-2401 binds to all three cell lines tested in a dose-dependent manner, with a RO_50_ of 10 nM for SK-CO-1 (high CEACAM5-expression), 20 nM for SNU-C1 (intermediate CEACAM5-expression), and 30 nM for LS174T (low CEACAM5-expression). Thus, an inverse correlation (R^2^ = 0.995, *P*=0.0147) between the CEACAM5-level and the RO_50_ of NILK-2401 was observed (**Supplementary Figure S4**).

#### Antibody-dependent cellular phagocytosis (ADCP)

**Figure 3:**
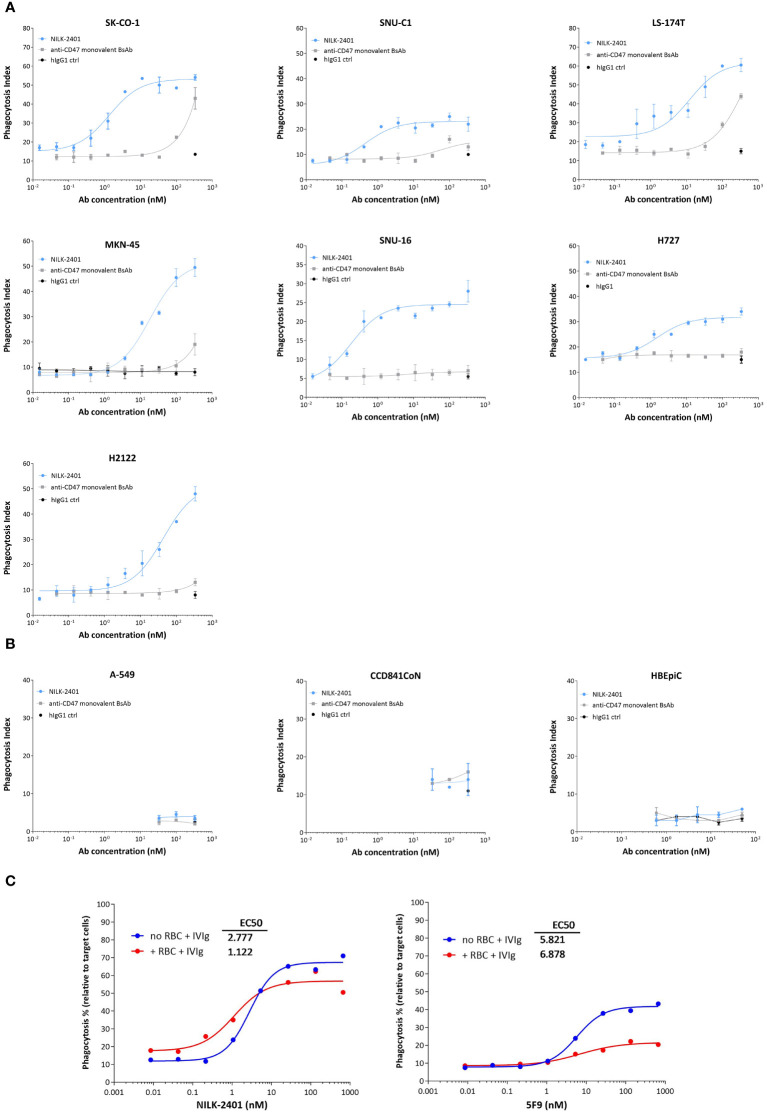
Phagocytosis activity of NILK-2401. Dose-dependent phagocytic activity of NILK-2401 towards **(A)** CEACAM5-expressing cancer cell lines SK-CO-1, SNU-C1, LS174T, MKN-45, SNU-16, H727, and H2122. **(B)** Absence of phagocytosis was observed for the CEACAM5-negative cell line A549 and to CCD841CoN, isolated from normal colon, as well as HBEpiC, isolated from normal bronchial tissue. **(C)** Concentration-response curves of phagocytosis induced by NILK-2401 (left panel) and 5F9 control antibody (right panel) in the presence of red blood cells (RBCs) and IgG excess (IVIg). Exemplary data for 1/4 donors with corresponding EC_50_ values are shown. Data show mean ± standard deviation. BsAb, bispecific antibody; ctrl, control.

NILK-2401 induced significant dose-dependent phagocytosis of all CEACAM5-positive tumor cell lines tested (**Figure 3A**) with EC_50_ ranging from 0.38 nM (SNU16) to 25.84 nM (H2122; **Supplementary Table S2**), as compared to an isotype control. The CD47 monovalent BsAb variant, which does not carry the CEACAM5-binding arm, induced significant killing only at the highest concentration tested of three out of the six cell lines tested (SK-CO-1, *P*=0.0011; MKN-45, *P*=0.0001; LS174T, *P*=0.0225), demonstrating the requirement of co-engaging CEACAM5 and CD47. NILK-2401 induced phagocytosis of cancer cell lines expressing different CEACAM5 levels, but no clear correlation between EC_50_ and the CEACAM5-density on tumor cells was observed (R^2^ = 0.2244). As expected, NILK-2401 did not induce phagocytosis of the CEACAM5-negative cell line A549, or primary epithelial cells, i.e., CCD841CoN and HBEpiC (**Figure 3B**).

In addition, we tested the impact of different concentrations of sCEACAM5 on the phagocytosis induced by NILK-2401. Results showed no statistical difference in ADCP activity with any of the cell lines tested, i.e., SK-CO-1, MKN-45 cells or H2122 cells (**Supplementary Figure S2B**).

Finally, we tested whether a potential CD47-sink effect, mimicked by the addition of an excess of CD47-expressing red blood cells (RBCs), could impact the phagocytosis induced by NILK-2401. An excess of 200-fold of RBCs, in comparison to the number of MKN-45 target cells, did not or only marginally impact ADCP of tumor cells induced by NILK-2401, as shown by similar E_max_ (67% without RBCs vs. 57% with RBCs) and EC_50_ (2.777 nM without RBCs vs. 1.122 nM with RBCs). In contrast, the bivalent anti-CD47 5F9 mAb, despite a weak property at inducing phagocytosis (EC_50_ of 5.821 nM and E_max_ of ≈40%), due to its hIgG4 backbone, is further impaired by the presence of RBCs (EC_50_ of 6.878 nM and E_max_ of ≈20%; **Figure 3C**). In summary, when erythrocytes were added, the ADCP potency of NILK-2401 was not impacted while that of 5F9 was suppressed.

#### Antibody-dependent cellular cytotoxicity

**Figure 4:**
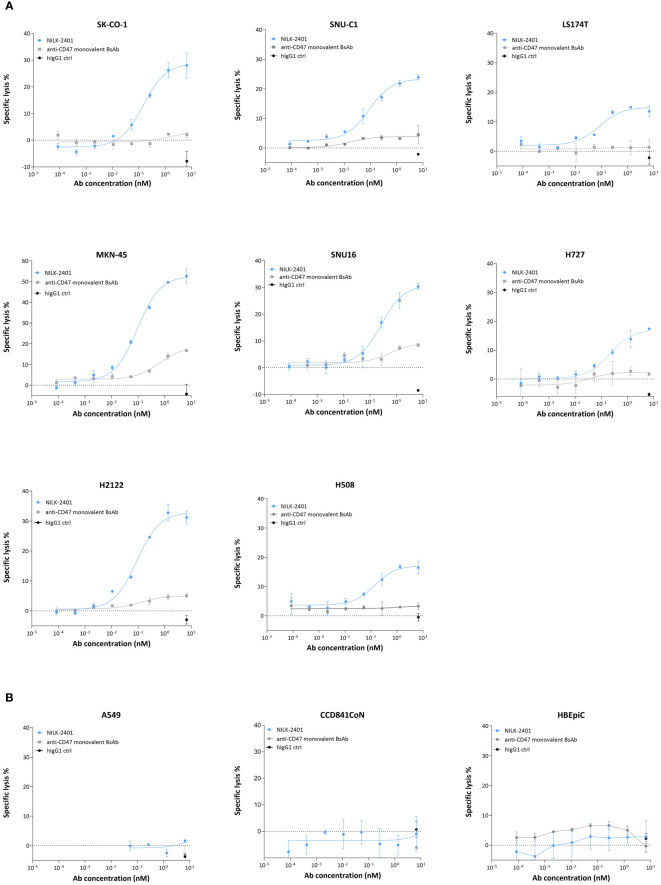
ADCC activity of NILK-2401. Dose-dependent ADCC activity of NILK-4201 towards **(A)** CEACAM5-expressing cancer cell lines SK-CO-1, SNU-C1, LS174T, MKN-45, SNU-16, H727, and H2122. **(B)** Absence of killing was observed for the CEACAM5-negative cell line A549 and CCD841CoN, isolated from normal colon, as well as HBEpiC, isolated from normal bronchial tissue. Effector (PBMCs): target ratio is 50:1. Exemplary data for one out of at least five donors. Data show mean ± standard deviation.

NILK-2401 induced dose-dependent antibody-dependent cellular cytotoxicity (ADCC) of all CEACAM5-positive tumor cell lines tested (**Figure 4A**), with EC_50_ ranging from 0.04 nM (LS174T) to 0.25 nM (SNU-16; **Supplementary Table S2**). The maximum killing induced by NILK-2401 was significantly higher as compared to the human IgG1 control antibody for all cell lines. The CD47 monovalent BsAb variant induced a very poor but significant killing compared to the IgG1 control at the highest concentration tested (SNU-C1, *P*=0.0003; SNU16, *P*=0.0023; H2122, *P*=0.0076; H727, *P*<0.0001; SK-CO-1, *P*=0.0002; MKN-45, *P*<0.0001; H508, *P*=0022; LS174T, *P*=0.0077). No clear correlation between EC_50_ and CEACAM5-density on tumor cells was observed (R^2^ = 0.00122). In contrast to the efficient killing of CEACAM5-positive tumor cells, NILK-2401 did not induce ADCC of the CEACAM5-negative cell line A549 or of primary epithelial cells (**Figure 4B**), which further supports the selectivity of this BsAb to CEACAM5-positive tumor cells.

When sCEACAM5 was added, a significant impact on NILK-2401-induced ADCC activity was observed for SK-CO-1, with the highest tested concentration of sCEACAM5 (0.1 µg/mL) rendering a 4-fold increase in EC_50_ as compared to the assay condition without sCEACAM5 (**Supplementary Figure S2C**). The two lower concentrations also showed a decreasing trend in NILK-2401 ADCC activity, but changes were not statistically significant. With MKN-45 cells, the EC_50_ was shifted, statistically non-significantly, by a factor of 2.5 and 3 for 0.05 and 0.1 µg/mL sCEACAM5, respectively. In case of H2122 cells, sCEACAM5 at 0.1, 0.05 and 0.02 µg/mL caused a statistically non-significant increase of EC_50_ by 3-, 2.8- and 1.8-fold, respectively, in comparison to the condition without sCEACAM5.

#### Complement-dependent cytotoxicity

Despite harboring a wildtype hIgG1 Fc-part, NILK-2401 did not show complement-dependent cytotoxicity (CDC) inducing activity in presence of the pool of human sera, used as source of complement (data not shown).

### *In vitro* safety profile

**Figure 5:**
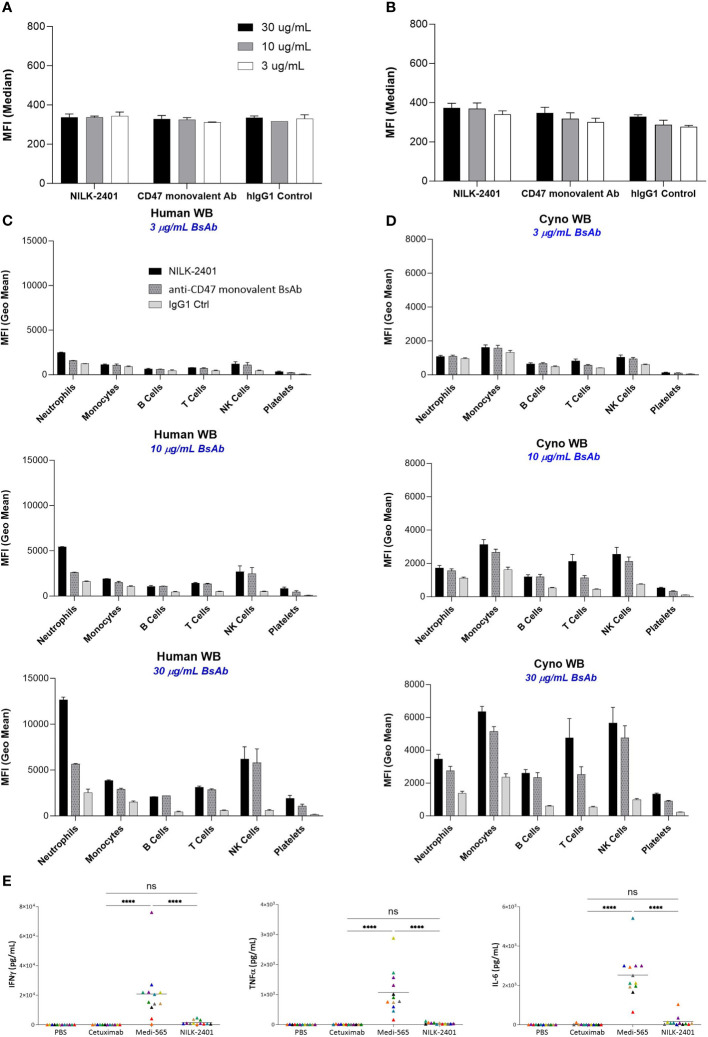
NILK-2401 binding to various cell populations from human and cynomolgus whole blood as well as cytokine release. Average MFI values for NILK-2401 binding to **(A)** human and **(B)** cynomolgus red blood cells (RBCs) as well as NILK-2401 binding (black bars) to neutrophils, monocytes, B-cells, T-cells, NK cells, and platelets from **(C)** human (n=2) and **(D)** cynomolgus (n=4) whole blood (WB) samples, compared to anti-CD47 monovalent control (ctrl) Ab (dashed grey bars) and IgG1 isotype control (light grey bars). Antibodies were tested at 3 µg/mL, 10 µg/mL, or 30 µg/mL. See also **Supplementary Table S3**. **(E)** Cytokine release profile of NILK-2401 using human whole blood from 12 healthy donors. Following 24 h of incubation with 330 nM of NILK-2401, Cetuximab or Medi-565 (330 nM), IFNγ (top panel), IL-6 (middle panel), and TNFα (bottom panel) were measured in supernatants using MSD technology. Each individual color point represents one donor. Phosphate-buffered saline (PBS), anti-EGFR mAb Cetuximab and CEACAM5xCD3 BiTe Medi-565 were used as controls. A two-way ANOVA test was performed for statistical significance. ****P<0.0001. BsAb, bispecific antibody; ns, not significant. Data show mean ± standard deviation.

As shown above, no binding to or killing of CEACAM5-negative cells or primary epithelial cells was observed *in vitro* with NILK-2401. Specificity of binding to CEACAM5 and CD47, and thus likelihood of absence of off-target effects, was likewise addressed using PEAK-cells expressing different CEACAMs (**Supplementary Figure S1**) and analysis of whole blood samples (**Figures 5A–D**, **Supplementary Table S3**).

#### Binding in human and cynomolgus whole blood

Results showed weak binding of NILK-2401 to human RBCs, similar to those of the anti-CD47 monovalent control and the hIgG1 control Abs, for all concentrations tested (30, 10 and 3 µg/mL). These data demonstrated the absence of specific binding of NILK-2401 to human RBCs (**Figures 5A, B**, **Supplementary Table S3**). No or only very weak specific binding of NILK-2401 was detected to the various other cell sub-populations in human and cynomolgus monkey blood at the lowest concentration tested (i.e., 3 µg/mL). The binding obtained on B cells, T-cells, NK-cells, monocytes, and platelets with NILK-2401 tested at 10 or 30 µg/mL was not increased compared to that of the anti-CD47 monovalent control antibody. A slightly higher level of binding on human neutrophils was detected with NILK-2401 as compared to the anti-CD47 monovalent control, but not on cynomolgus neutrophils (**Figures 5C, D**).

#### Erythrophagocytosis

Due to the weak binding to RBCs observed in the circulation, we assessed whether NILK-2401 would induce phagocytosis of RBCs. In presence of 1.5 mg/mL of human IgG, no erythrophagocytosis was induced by NILK-2401, up to the highest antibody concentration tested (300 μg/mL). In contrast, B6H12, a chimeric human IgG1 anti-CD47 mAb, induced erythrophagocytosis with an average EC_50_ of 1.73 nM and an average percentage of phagocytosis top plateau of 74% (**Supplementary Figure S5A**). These data demonstrate a negligible or no impact of NILK-2401 on erythrocytes *in vitro*, which can be explained by the low binding of the low-affinity anti-CD47 arm.

#### Hemagglutination

Due to the monovalent binding to CD47, which will not allow cross-linking among RBCs, NILK-2401 did not induce hemagglutination in human whole blood, up to the highest tested concentration of 100 μg/mL (**Supplementary Figure S5B**).

#### Platelet activation

Upregulation of CD62P on platelets was used as surrogate to assess platelet activation. Induction of CD62P expression was only observed when using the positive controls, i.e., following incubation with adenosine diphosphate or anti-CD9, but not by NILK-2401 (**Supplementary Figure S5C**).

#### Whole blood cytokine release

Considering the weak binding detected to hCEACAM3, which is expressed on neutrophils, the potential of NILK-2401 to induce inflammatory cytokines, i.e., IFNγ, TNFα, and IL-6 was evaluated (n=12 healthy donors). Although NILK-2401 could induce a slight but not significant (P>0.8) increase above the negative control, Cetuximab, in the measured cytokines, levels were significantly lower than the positive control, MEDI-565, a bispecific T-cell engager targeting CEACAM5 and CD3 for which cytokine release is to be expected (33), for all three cytokines. Mean levels for IL-6 were 176.9 vs. 2524 pg/mL (P<0.0001), TNFα 48.38 vs. 1075 pg/mL (P<0.0001), and IFNγ 2641 vs. 20,790 pg/mL (P<0.0001; **Figure 5E**).

### *In vivo* activity

**Figure 6:**
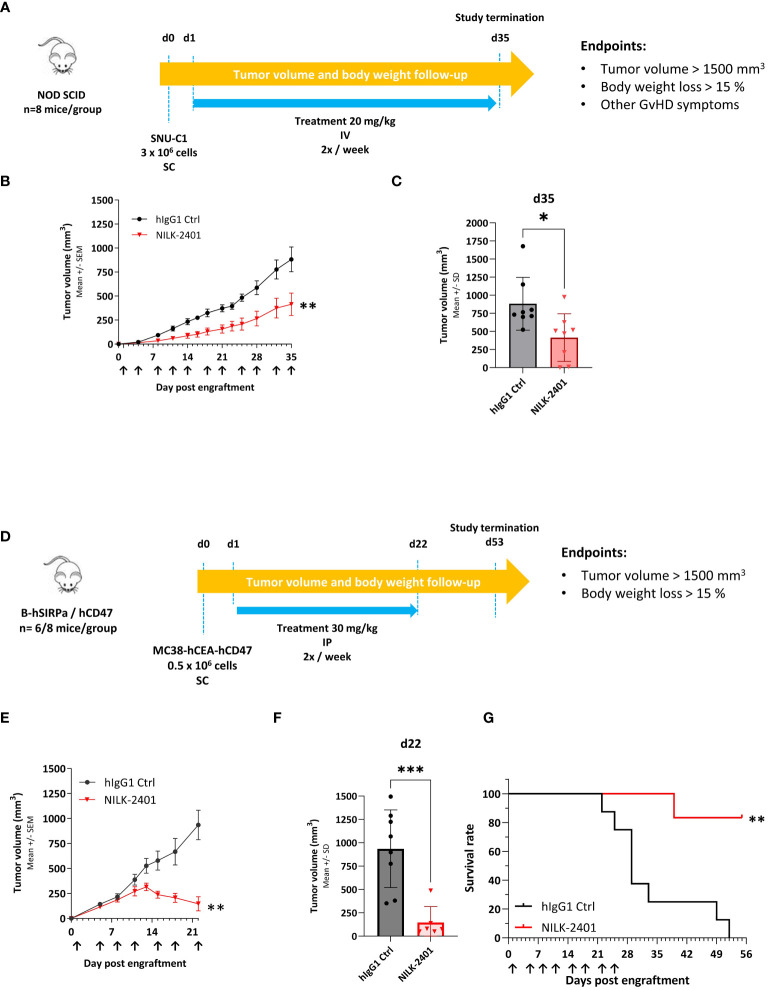
NILK-2401 *in vivo* anti-tumor activity. **(A)** SNU-C1 xenograft model. NOD SCID mice were subcutaneously (SC) injected with 3 x 10^6^ SNU-C1 tumor cells (d0) and received twice a week intravenous (IV) injection of 20 mg/kg of the specified treatment (n=8 mice per group). **(B)** Tumor volume average over time. Data show mean ± SEM. **(C)** Tumor volume on d35 post engraftment; arrows indicate days of treatment injections. Data show mean ± SD. Statistical analysis was performed on the area under curve (AUC, between d0 and d35) and the tumor volume average using unpaired t-test. **(D)** Transgenic mouse model. hCD47/hSIRPα transgenic mice were SC injected with 0.5x10^6^ MC38-hCEA-hCD47 tumor cells (d0) and received twice a week intraperitoneal injection of 30 mg/kg of NILK-2401 (n=6) or vehicle control (ctrl; n=8) starting from d1. **(E)** Tumor volume average over time. Data show mean ± SEM. **(F)** Tumor volume 22 days post engraftment. Data show mean ± SD. Arrows indicate days of treatment injections. **(G)** Survival curve till the end of the experiment (day 53 post engraftment). For statistical analysis, unpaired t-test was performed on the area under curve (AUC at d22; **(E)** and the tumor volume average **(F)**. A Log-rank (Mantel-Cox) test was performed for survival analysis **(G)**. *P<0.05, **P<0.01, ***P<0.001. SD, standard deviation; SEM, standard error of the mean.

The *in vivo* anti-tumor activity of NILK-2401 was investigated in a mouse xenograft model, using SNU-C1 as target cells, as well as in a syngeneic model using human SIRPα/human CD47 transgenic mice with MC38 transfected with human CEACAM5 and human CD47 (**Figure 6**).

NOD SCID mice were exposed to 20 mg/kg NILK-2401 IV twice weekly vs. control IgG1. NILK-2401 significantly delayed tumor growth, with a TGI of 53% at d35, i.e., 1^st^ day of euthanasia due to tumor volume >1500 mm^3^ (**Figures 6A–C**).

In hSIRPα/CD47 transgenic mice, NILK-2401 treatment at 30 mg/kg IP twice/week significantly suppressed tumor growth of MC38-hCEACAM5/hCD47, with a TGI of 84.3% at d22 (i.e., 1^st^ day of euthanasia due to tumor volume >1500 mm^3^; **Figures 6D–F**). Prolonged treatment with NILK-2401 resulted in almost complete tumor regression followed by relapse of the tumors several days after stopping the treatment. At the end of the experiment (52 days post engraftment), all isotype control-treated mice were euthanized due to high tumor volume, whereas NILK-2401-treated mice showed a survival rate of 83% (**Figure 6G**).

### Pharmacokinetics

The PK profile of NILK-2401 was evaluated in cynomolgus monkeys as part of a non-GLP single-dose study and in Tg32 FcRn humanized mice.

The anti-CEACAM5 arm is not cross-reactive to cynomolgus CEACAM5, while the anti-CD47 arm of NILK-2401 does recognize the cynomolgus target with similar affinity (see above).

**Figure 7:**
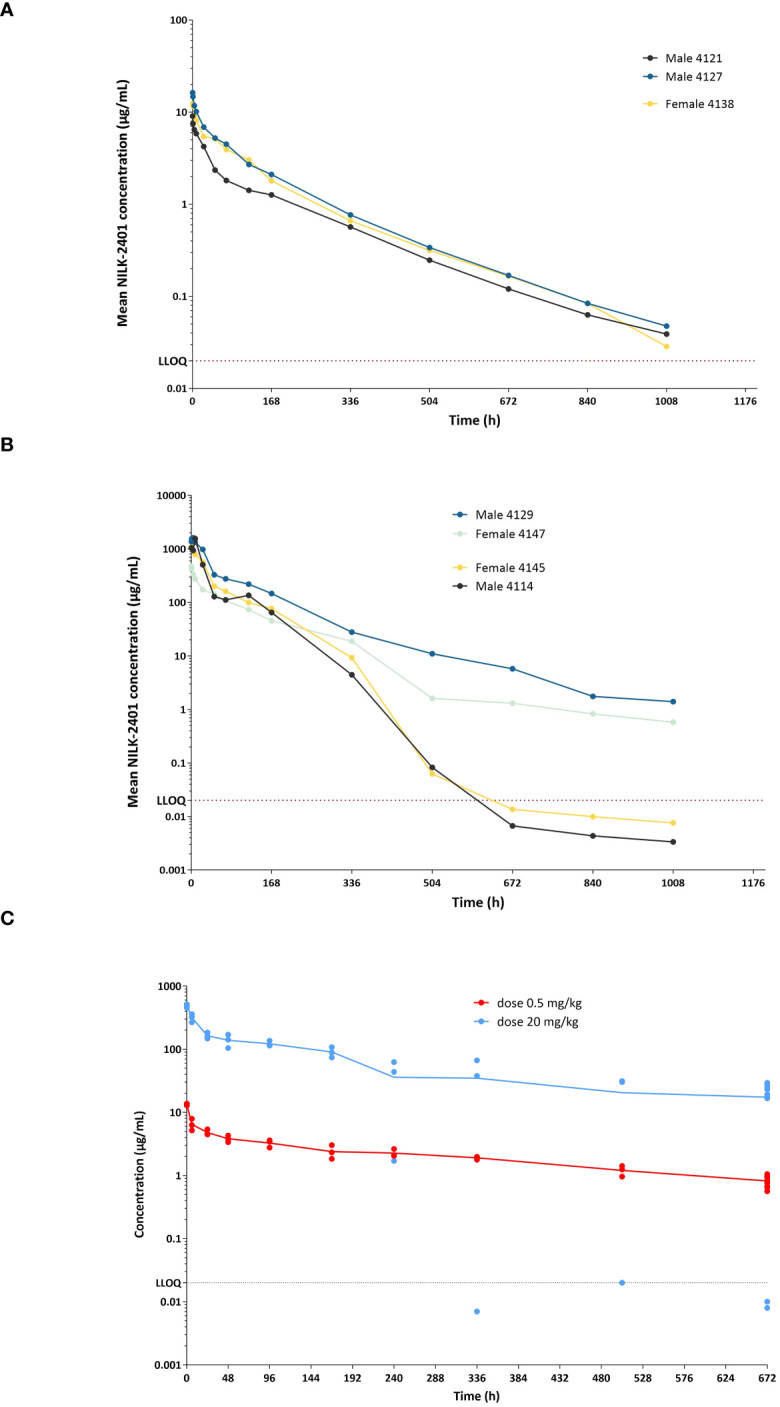
NILK-2401 concentrations versus time in cynomolgus monkeys and Tg32 human FcRn mice. Male and female cynomolgus monkeys received a single IV administration at **(A)** 0.5 mg/kg or **(B)** 20 mg/kg, respectively. NILK-2401 serum concentrations were quantified at different time-points following the IV injection using a fit-for-purpose PK assay. Each line corresponds to a different animal, with full lines and dashed lines corresponding to female and male animals, respectively. The horizontal black dashed line corresponds to the LLOQ (lower limit of quantification). **(C)** PK profile was also analyzed in Tg32 human FcRn mice following a single injection of NILK-2401 at 0.5 mg/kg (light blue dots) and 20 mg/kg (red dots). PK parameters are summarized in **Supplementary Tables S4** and **S5**.

Following IV administration at 0.5 mg/kg (**Figure 7A**, **Supplementary Table S4**), NILK-2401 concentrations decreased steadily until the last time point (i.e., 1,008 hours), although the decrease seemed slightly higher in one monkey between 0.25 and 168 hours. After 168 hours, elimination was similar across all animals.

After IV administration at 20 mg/kg (**Figure 7B**, **Supplementary Table S4**), concentrations decreased steadily till the last time point in two monkeys. In the two other monkeys, concentrations declined steeply after 168 hours and were below the limit of quantification after 672 hours. Concentrations during the first 24 hours in three monkeys were higher compared to the other monkey (range: 508-1618 μg/mL vs. 173-473 μg/mL).

NILK-2401 PK seemed to be more than dose-proportional for C_max_ and AUC_0-inf_. No gender differences in PK were observed.

PK data were confirmed in Tg32 human FcRn mice (**Figure 7C**, **Supplementary Table S5**), although several values were flagged because they did not fulfill at least one of the conditions for reliable derivation. NCA of the mean PK data showed a dose-related increase in C_max_ and AUC_last_. Clearance (CL) seemed slightly higher after injection at 0.5 mg/kg compared with the 20 mg/kg dose level (0.283 vs. 0.183 and 0.203 mL/h/kg, respectively, depending on the inclusion or exclusion of outliers).

### Tolerability

Doses of 0.5 and 20 mg/kg, given as single IV administration, were well tolerated over the 6-week observation period (data not shown). No abnormalities in clinical signs, including observation of the injection site, and food intake were observed, and no toxicologically meaningful changes were present in body weights, safety pharmacology (i.e., neurological behavior, ECG, blood pressure and respiratory function), clinical pathology (i.e., hematology, clinical chemistry, and coagulation) and at post-mortem examinations.

Mortality was observed in one low-dose female monkey immediately after administration. No clinical signs were observed before death and no grossly abnormal observations were recorded at necropsy. Following histological examination of the full list of collected tissues, a clear cause of death was not defined. However, based upon the single incidence of mortality and since no signs of toxicity were noted in other animals, the death of this animal was considered unlikely to be related to NILK-2401 treatment.

Likewise, NILK-2401 was well tolerated in human FcRn Tg32 mice (data not shown); no clinical signs, no weight loss, or mortality were observed following injections of 0.5 and 20 mg/kg NILK-2401, respectively.

### Immunogenicity assessment

The immunogenicity potential of NILK-2401 was evaluated by quantification of the pre-existing NILK-2401-reactive CD4 T-cell repertoire in the blood of 14 normal donors with diverse HLA molecules in parallel to controls including Adalimumab (anti-TNFα), Trastuzumab (anti-HER2 mAb), and KLH. The therapeutic antibodies Adalimumab and Trastuzumab are described to be potentially non-immunogenic and immunogenic proteins, respectively (43, 44), while KLH is a highly immunogenic T-cell dependent antigen (45).

Across the 14 KLH responding donors, nine donors responded to Adalimumab, and exhibited a mean response rate of 0.2 specific CD4 T-cells per million. Six donors responded to Trastuzumab and exhibited a mean response of 0.1 specific CD4 T-cells per million, which is at the cut-off between low and moderate T-cell response. Five donors responded to NILK-2401 and exhibited a mean response of 0.07 specific CD4 T-cells per million. Overall, these results show that NILK-2401 has a T-cell response rate in the category of low risk of immunogenicity (**Supplementary Figure S6**).

### NILK-2401 plus NILK-2301 combination activity

**Figure 8:**
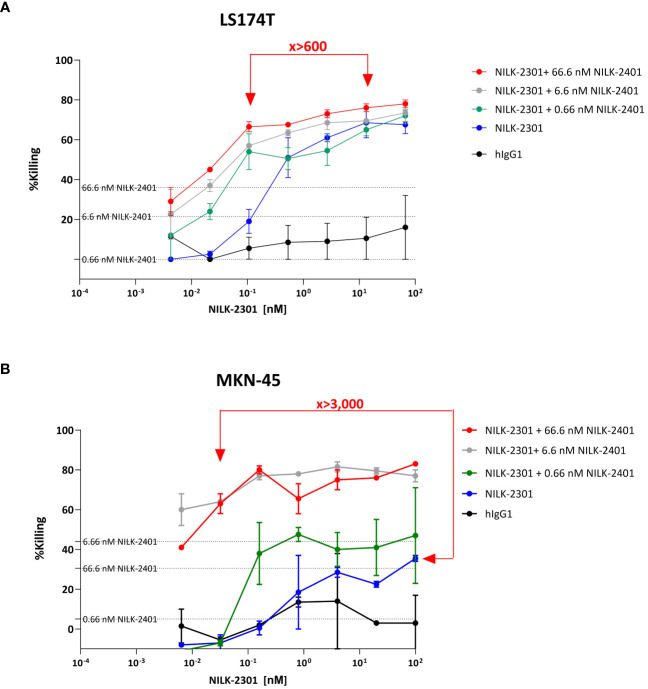
NILK-2401 plus NILK-2301 combination activity. “Mixed killing assay” using **(A)** LS174T and **(B)** MKN-45 as target cells, respectively. Cells were exposed to NILK-2301 alone (blue curve) or NILK-2301 plus NILK-2401 at 0.66 nM (green curve), 6.6 nM (grey curve), or 66.6 nM (red curve), respectively. Cells without treatment (pink curve) or those treated with human IgG1 control antibody (hIgG1; black curve) were used as comparators. NILK-2401 can boost NILK-2301 activity, e.g., equivalent activity of NILK-2401 plus NILK-2301 combination therapy at >600-fold (LS174T) to >3,000-fold MKN-45) lower doses compared to NILK-2301 monotherapy, i.e., ≈0.02 vs. 13.3 and, ≈0.03 vs 100 nM, were found. Data show mean ± SEM.

When combining two different mechanisms of action, i.e., inhibition of CD47 and re-targeting of T-cells to the tumor, equivalent activity of NILK-2401 plus NILK-2301 therapy at >600-fold (LS174T) to >3,000-fold (MKN-45) lower doses compared to NILK-2301 monotherapy was observed, i.e., ≈0.02 vs. 13.3 and, ≈0.03 vs. 100 nM, respectively. Combination with NILK-2401 can thus boost NILK-2301 activity (i.e., Emax) by 15 to 250% (**Figure 8**).

## Discussion

Here we describe a novel CEACAM5xCD47 BsAb, termed NILK-2401, constructed using the κλ body platform. This format is assembled by co-expressing one heavy chain and two different light chains, one κ and one λ, resulting in a BsAb that fully retains the sequence and architecture of human antibodies (40). The κλ format presumably conveys low antigenicity and concomitantly lower potential for induction of neutralizing anti-drug antibodies (ADAs). Clinical evidence for this is given by ongoing clinical trials involving other BsAbs based on the same κλ body format: TG-1801 is a CD19xCD47 BsAb (42) that is being tested in Phase I clinical trials in patients with B-cell malignancies (NCT03804996 and NCT04806035) both in monotherapy as well as in combination (46) and NI-1801, a MSLNxCD47 BsAb (47), that is being tested in a Phase I clinical trial in patients with MSLN-positive solid tumors (NCT05403554) (48). Both molecules have the same CD47-targeting arm (K2) as NILK-2401. In agreement, the results of the immunogenicity assessment showed that NILK-2401 has a T-cell response rate in the category of low risk of immunogenicity, with the T-cell response rate of NILK-2401 being lower than that of Trastuzumab with known low immunogenicity potential (44), when tested using the same CD4 T-cell donors. Overall, this suggests that NILK-2401 is likely to have a low risk of immunogenicity in human beings. In contrast, the high variability observed in the PK concentration-time profiles in monkeys is likely caused by immunogenicity (e.g., ADA-formation), as expected in such an experimental setting (49). Outliers, likely attributable to ADAs as well, were also found in Tg32 mice, and several values (e.g., CL) were flagged because they did not meet at least one of the conditions for reliable derivation. Although CL might be slower in Tg32 mice, the CL values calculated for NILK-2401 in the present study were nevertheless consistent with the range previously reported for 17 mAbs in wild type mice (0.13-2.19 mL/kg, median: 0.36 mL/kg) (50).

NILK-2401 targets human CEACAM5 with a high-affinity and human CD47 with an optimized arm of intended lower affinity. Additionally, NILK-2401 contains an unmodified (i.e., wildtype) IgG1 Fc region and can therefore mediate effector functions through binding to FcγRs. This is in contrast to most molecules in clinical development which have an inactive/silent Fc part (51) not able to recruit either the complement system or phagocytes and thus requiring combination treatment with compounds that deliver necessary co-stimulating, pro-phagocytic signals, e.g., “active”, tumor-opsonizing monoclonal antibodies to enhance phagocytosis.

The higher affinity of NILK-2401 binding to human CEACAM5 enables the selective CD47-blockade on CEACAM5-expressing cells by simultaneous binding to CEACAM5 and CD47, whereas the binding to CD47^+^CEACAM5^-^ cells (e.g., RBCs and platelets) is limited by the low affinity of the anti-CD47 arm. The low affinity interaction driven by the anti-CD47 arm only is, based on our own research, insufficient for stable binding on CEACAM5-negative cells. In contrast, the high-affinity binding mediated by the anti-CEACAM5 arm induces the co-engagement of CD47 resulting in a “guided inhibition” of the CD47-SIRPα interaction between CD47 on CEACAM5-positive tumor cells and SIRPα on e.g., macrophages and natural killer cells. As shown by others and us (20, 47, 52–55), this targeted approach has the advantage of avoiding potential side effects due to the otherwise arbitrary blockade of CD47 on normal cells, as observed for other CD47-targeting approaches, with anemia and thrombocytopenia being the most frequent adverse events associated with CD47 mAbs (51). By avoiding this sink, we also circumvent the need for a loading/priming dose which is required for other anti-CD47 targeting agents, including magrolimab (Hu5F9-G4) (8). In agreement with the mechanism of action of NILK-2401, only a minimal, not dose-dependent decrease in RBCs, hemoglobin, and hematocrit (10-15%) was recorded on day 3 in both sexes at all doses in the cynomolgus tolerability study, followed by a slight to marked increase in reticulocyte count (2.4-9.0-fold) on day 15. Normalization in the red cell compartment was recorded starting from day 29 and up to day 43 in all treated animals. Only one animal at 20 mg/kg showed a slight decrease in platelet count (35%; within range) on day 3, which normalized by day 15. Overall, changes in the red cell and platelet compartments were considered secondary to the repeated blood sampling, and not treatment related. Also, ADCP of CEACAM5^+^/CD47^+^ tumor cells induced by NILK-2401 was not impacted by the presence of a CD47 sink (i.e., presence of CD47-positive RBCs). NILK-2401 was unable to elicit CDC *in vitro*. Nevertheless, we cannot exclude the possibility that CEACAM5-positive malignant cell lines use membrane-associated complement regulatory proteins to inhibit NILK-2401 mediated CDC *in vitro*.

While cynomolgus monkeys and Tg32 mice were deemed relevant for hazard identification, given the lack of full cross-reactivity (and thus pharmacological activity), as the anti-CEACAM5 arm is not cross-reactive with cynomolgus and no CEACAM5 orthologs exist in rodents or other species commonly used in nonclinical safety evaluations (56), no standard toxicity studies were considered justified in these species.

Although NILK-2401 can bind weakly to CEACAM3 (K_D_≈700 nM vs. ≈4 nM to CEACAM5), a receptor expressed in neutrophils (25, 57–59), based on *in vitro* data, this was not associated with an increased risk of cytokine release in whole blood.

Combination with a CEACAM5-targeted T-cell engager (NILK-2301 (60)) resulted in increased activity, i.e., EC_50_ was reduced by a factor of 10-100, and at the same time the required dose of the T-cell engager was reduced. In summary, by combining two different mechanisms of action that address both the innate as well as the adaptive immune system, we anticipate that this will result in a lower likelihood of side effects like cytokine release syndrome in patients with CEACAM5-expressing solid tumors treated with bispecific T-cell engagers like NILK-2301 (60).

In conclusion, NILK-2401 combines promising preclinical activity and safety with format-associated lower probability of ADA-generation. NILK-2401 is planned to enter clinical testing in patients with CEACAM5-expressing solid cancers.

## Data availability statement

The original contributions presented in the study are included in the article/**Supplementary Material**. Further inquiries can be directed to the corresponding author.

## Ethics statement

Anonymized human samples as by-products (i.e., buffy coats) were obtained from healthy individuals from the Établissement Franç̧ais du Sang (EFS, Rungis, France) for immunogenicity experiments and the Hoĉpitaux Universitaires de Genève (HUG) for all other experiments involving primary cells. All studies were approved by the ethics committees of the respective institutions.  As no other sample types were used and no samples were specifically collected for our study, no additional specific ethics vote and no further written informed consent for participation was required from the participants or the participants’ legal guardians/next of kin in accordance with the national legislation (France, Switzerland), European and institutional requirements.

The animal study was approved by the Cantonal Veterinary Office Geneva. The approval numbers are: SNU-C1 xenograft model in NOD SCID (#GE134), hCD47/hSIRPa transgenic mice with MC38-hCEA-hCD47 tumor cells (#GE299), and Tg32 human FcRn mice (#GE36). The study was conducted in accordance with the local legislation and institutional requirements.

## Author contributions

AS: Writing – original draft, Writing – review & editing. VB: Writing – original draft, Writing – review & editing. VM: Writing – original draft, Writing – review & editing. BD: Writing – original draft, Writing – review & editing. FR: Writing – original draft, Writing – review & editing. LC: Writing – original draft, Writing – review & editing. AV: Writing – original draft, Writing – review & editing. NB: Writing – original draft, Writing – review & editing. ER: Writing – original draft, Writing – review & editing. KM: Writing – original draft, Writing – review & editing. SS-P: Writing – original draft, Writing – review & editing. CB: Writing – original draft, Writing – review & editing. CM: Writing – original draft, Writing – review & editing. FJ-B: Writing – original draft, Writing – review & editing. YP: Writing – original draft, Writing – review & editing. DH: Writing – original draft, Writing – review & editing.
