# Supplementary material for: Targeting CEACAM5-positive solid tumors using NILK-2401, a novel CEACAM5xCD47 κλ bispecific antibody
Source: Front Immunol. 2024 Apr 24;15:1378813. doi: 10.3389/fimmu.2024.1378813 (PMC11076849; doi:10.3389/fimmu.2024.1378813)
Supplement: Supplementary file 1 [file DataSheet_1.pdf]

DATA SUPPLEMENT

SUPPLEMENTARY METHODS

Affinity to CEACAM5 and CD47

Protein A biosensors (Sartorius) were loaded with two different batches of NILK-2401 at a concentration of 0.5 µg/mL for 300 s and a loading signal between 0.7 and 0.85 nm was obtained. A 2x serial dilution of human recombinant CEACAM5 and shed human CEACAM5 (Bio-Rad) was prepared in KB buffer (Sartorius), starting at 100 nM for the first replicate and 50 nM for the second, on seven different concentrations, the eighth well of the column corresponding to a blank buffer for reference well subtraction. The association and dissociation steps were monitored for 600 s each. Finally, a regeneration step was applied to reuse biosensors for another interaction. Experiments were performed using an Octet RED96 instrument (Sartorius).

Data were processed and analyzed using the Data Analysis software (Fortebio). Both association and dissociation steps were fitted using a global 1.1 full fitting model.

Affinity measurement of the NILK-2401 kappa arm for human and cynomolgus CD47 was performed at GenScript (China). The biotinylated human CD47 soluble recombinant protein was captured on a streptavidin coated sensor chip (Cytiva). A concentration series of the BsAb, starting at 800 nM (human) and 25 nM (cynomolgus), was injected over the surface, with regeneration of the surface between each injection. Two (human) or three (cynomolgus) experiments at different loading levels (50 and 250) were performed using a Biacore T200 instrument.

Data were double referenced (running buffer injection and BsAb injections on a reference surface) to subtract potential unspecific background signals. Curves were analyzed applying a steady state fitting model.

Epitope binning

Epitope binning was performed by assessing the binding of anti-CEACAM5 mAbs to recombinant CEACAM5 protein in the presence of reference antibodies. Six mAbs previously described as binding to different epitopes of human CEACAM5 were produced as human IgG1 and/or mouse IgG2a and used as reference antibodies: 1) mAb derived from SM3E (patent US20050147614A1, binding to the N-terminal domain); 2)

mAb derived from MEDI-565 (WO2016036678A1, binding to the A2 domain); 3) mAb derived from Mab2_VLg5VHg2 (EP3199552A1, binding to the A3B3 domain); 4) mAb derived from CH1A1A-2F1 (US20120251529); 5) mAb derived from variant 1 described in patent WO2017055389 (binding to the A3 domain); and 6) mAb derived from hMN14 (US 2002/0165360A1, binding to the B3 domain).

Epitope mapping

The binding epitope of the CEACAM5-targeting arm (termed AC100) was determined by sequential mutagenesis. Full length recombinant human CEACAM5 mutants (in the N-terminal domain) were designed according to the alignment of human and cynomolgus CEACAM5. Mutants and wild type sequences were cloned into pEAK8 vector (Edge Bio). The constructs were then transfected and expressed at the surface of PEAK cells (ATCC). Expression level of all constructs was confirmed using a benchmark antibody which binds both human and cynomolgus CEACAM5. AC100 mAb binding was analyzed by flow cytometry.

CD47-SIRPα interaction

The detection of the binding of soluble SIRPα to human CD47 expressed at the surface of MKN-45 was used for the assessment of the CD47/SIRPα ligation blocking activity of NILK-2401. MKN-45 cells, expressing both CD47 and CEACAM5 (Supplementary Table S1), were stained with carboxyfluorescein succinimidyl ester (CFSE) violet (Invitrogen) and 3,000 stained cells per well were seeded in a 384 optical well plate (Costar) and incubated for 50 min with increasing concentrations of NILK-2401 (1.9 pM to 333 nM, in quadruplicates). SIRPα mouse-Fc premixed with anti-mouse IgG-Fc AF647 coupled Ab (Jackson ImmunoResearch, diluted 1:2,000) was added at 50 ng/mL final concentration. After an incubation of 3 h 30 min, plates were acquired using a CellInsight CX5 imager (ThermoFisher Scientific) and fluorescence signals emitted by the detected bound SIRPα were recorded. Mean fluorescence intensity (MFI) signals were plotted and IC_{50} calculated using Prism software (GraphPad).

NILK-2401 binding

Binding to other CEACAM family members was assessed using transfected PEAK cells. In brief, the full-length DNA sequence coding for human CEACAM 1, 3, 4, 6, and 8 proteins was cloned into pEAK8 vector (Edge Bio)

and transfected into PEAK cells (ATCC). Transiently expressing cells were used 48h-72h post-transfection, following protein expression level determination by flow cytometry using commercially available CEACAM-specific antibodies (Supplementary Table S6).

NILK-2401 binding to CEACAM5-expressing cell lines compared to CEACAM5-negative cells was tested at a dose range of 0.0128 to 200 nM using flow cytometry. The three highest concentrations were tested with CEACAM5-negative cells. To assess the impact of soluble CEACAM5 (sCEACAM) on NILK-2401 binding, 0.02, 0.05 and 0.1 µg/mL sCEACAM5 (Biorad) was added to selected experiments.
2.5×10^5 cells/well were centrifuged in a 96-well-V-bottom plate and incubated for 20 min at 4°C, with 100 µL of NILK-2401, anti-CD47 monovalent BsAb or isotype control, at a range of concentrations, starting from 200 nM as the highest concentration to 0.0128 nM as the lowest concentration, with 1/5 serial dilutions covering six concentration points, in phosphate-buffered saline (PBS) containing 2% (w/v) bovine serum albumin (BSA) as binding buffer. Cells were washed twice and 100 µL of secondary Ab (mouse anti-human IgG-Fc-PE, Southern Biotech; 100x diluted) was added and incubated for 20 min. After washing, cells were resuspended with 150 µL of binding buffer containing Sytox Blue (ThermoFisher Scientific; 5000x diluted). Cells were analyzed using a CytoFLEX flow cytometer (Beckman Coulter) and raw data extracted by using FlowJo software (BD Biosciences). Geometric MFI was extracted from the "live cell" gate and plotted against the concentrations of NILK-2401 and control antibodies using Prism software.

To assess the binding to corpuscular components in whole blood samples from healthy human donors (Centre de Transfusion Sanguine Genevois (CTS), Switzerland) and cynomolgus monkeys (BioPrim; two males and two females), directly labeled NILK-2401 was used. As a first step, NILK-2401 as well as an anti-CD47 monovalent control and human IgG1 control Abs were labeled with Alexa Fluor-488 (AF488 protein labeling kit, Life Technologies). Specific binding of AF488-labeled NILK-2401 was verified on SK-CO-1 cells. AF488-labeled Abs were tested at 30, 10 or 3 µg/mL (i.e., 200, 67 or 20 nM, respectively). The antibody staining panel to gate the various cell subsets included the following: mouse anti-human CD41a_PE, mouse anti-human CD56_PerCP-Cy5.5, mouse anti-human CD3_APC-Cy7, mouse anti-human CD14_BV421, mouse anti-human CD45_PE-Cy7, mouse anti-cynomolgus CD45_PE-Cy7, mouse anti-human CD16_BV605, mouse anti-

human CD10_APC (all from BD Biosciences), and mouse anti-human CD20_BV510 (BioLegend). Human or cynomolgus whole blood samples were added in 96-round bottom assay plates, previously filled with 20 µL of (10x) AF488-labeled BsAbs solutions and 20 µL of (10x) antibody staining panel mix and incubated for 30 min at 4°C.

To assess binding of AF488-labeled NILK-2401 on red blood cells (RBCs), 5 µL of whole blood incubated with antibodies were transferred to another plate and diluted 40-fold with PBS. Plates were centrifuged and supernatants discarded. Cells were resuspended with 150 µL/well of binding buffer, and AF488 MFI was acquired. The initial assay plate was treated twice with 200 µL/well FACS lysing solution (BD Biosciences) to remove RBCs and pellet leucocytes and platelets only. Finally, plates were resuspended with 70 µL/well binding buffer, duplicates were pooled, and fluorescence for both AF488-labeled BsAbs and surface staining antibodies was acquired.

Data were analyzed using FlowJo software, and binding of AF488-labeled Abs on the various cell subtypes was plotted and overlaid on histogram panels.

Receptor occupancy (RO)

NILK-2401 was tested in a dose range of 0.03 to 6000 nM with tumor cell lines LS174T and SNU-C1, and of 0.003 to 600 nM with SK-CO-1, for its ability to bind to both CEACAM5 and CD47 and saturate targets expressed on three different colorectal tumor cell lines, i.e., LS174T, SNU-C1, SK-CO-1, expressing different level of CEACAM5.

After incubation, cells were centrifuged and washed twice in cold binding buffer before addition of 100 µL of a mouse anti-human IgG-Fc-PE (Southern Biotech) secondary detection Ab. Cells were centrifuged and washed twice in cold binding buffer and then resuspended in 150 µL binding buffer containing Sytox Blue as viability marker (ThermoFisher Scientific). Cells were then subjected to flow cytometry using a CytoFLEX flow cytometer (Beckman Coulter). CytoFLEX raw data were extracted by using FlowJo software: Geometric mean from anti-mouse IgG-PE mean fluorescence intensity (MFI) was extracted from the "viable cell" gate and plotted against NILK-2401 concentrations using Prism software.

RO (i.e., saturation level of NILK-2401 targets expressed at the tumor cell surface) was calculated for each NILK-2401 concentration using the following formula:

RO% = (MFI at a tested NILK-2401 concentration / max MFI) x 100

Max. MFI is the highest MFI obtained among the range of NILK-2401 concentrations tested for respective cell lines.

Antibody-dependent cellular phagocytosis (ADCP)

To assess the impact of RBCs on ADCP, $30x10^6$ erythrocytes purified from human whole blood (Blood Transfusion Center, Geneva, Switzerland) were incubated with NILK-2401 at concentrations up to 100 µg/mL, in the presence of IgG excess, then mixed with 0.1 million MKN-45 tumor cells stained with carboxyfluorescein succinimidyl ester (CFSE) and 0.1 million PBMC-derived, differentiated macrophages. Following incubation, macrophages were stained an anti-CD14-APC antibody (eBioscience); non-engulfed RBCs were lysed (FACS™ lysing solution, BD). Flow cytometry was performed to determine ADCP for each antibody concentration, corresponding to the percentage of tumoral cells being engulfed by macrophages. A human IgG1 mAb was used as negative control, the anti-CD47 mAb 5F9-hIgG4 (Liu et al., 2015) as positive control, respectively (both produced in-house at LCB).

Data were analyzed using FlowJo software. The percentage of phagocytosis relative to target cells was determined as the percentage of CFSE/CD14 double positive cells within the target cell population. Dose-response curves were generated and EC_{50} values were determined using Prism software. A total of three experiments were performed, with a total of six donors analyzed.

Complement-dependent cytotoxicity (CDC)

CDC-induction in three CEACAM5-positive cell lines (HPAFII, MKN-45, and SK-CO-1) was investigated by using 20% normal human serum (NHS; Sigma-Aldrich) as a source of complement. Cells were diluted in their respective medium at $0.2.10^6$/mL and 50 µL of cell suspension was seeded/well. Afterwards, 25 µL of 4x concentrated Abs (i.e., anti-MHC-I mAb [positive control], human IgG1 [isotype control], NILK-2401) were added (50 µg/mL final concentration). After 15 min, 25 µL of NHS was spiked (20% final concentration) for 3 h and 24 h at 37°C. The number of viable cells was assessed by quantitation of the adenosin triphosphate

(ATP) present in the supernatant using the CellTiter-Glo® Luminescent Cell Viability Assay Kit (Promega) according to the manufacturer's protocol and a SpectraMax i3x luminometer (Molecular Devices). CDC was quantified and expressed as relative light units (RLU). The percentage of cell lysis was calculated by using the following formula:

Specific lysis% = 100 - (RLU test Ab - RLU background) / (RLU w/o Ab - RLU background) x 100%

A total of three experiments were performed.

Erythrophagocytosis

Erythrocytes purified from human whole blood were stained with CFSE and incubated with NILK-2401 at concentrations up to 300 µg/mL or B6H12 (produced in-house; positive control), in the presence of IgG excess, then mixed with human PBMC-derived, M-CSF differentiated macrophages from two different healthy donors at an effector to target ratio of 1:200 for 1 h at 37°C.

Following incubation, macrophages were stained with an anti-CD14-APC antibody (eBioscience); non-engulfed RBCs were lysed. Flow cytometry was performed to determine erythrophagocytosis for each antibody concentration. Data were analyzed using FlowJo software.

The percentage of erythrophagocytosis is defined as the percentage of macrophages that have engulfed at least one RBC. Dose-response curves were generated and EC_{50} were determined using GraphPad Prism software.

Hemagglutination

Whole blood samples from human healthy donors, collected in citrate anti-coagulant, were centrifugated and washed three times in PBS. RBCs were resuspended in blocking buffer (PBS + 2% BSA) and $3x10^6$ cells were added to round-bottom 96-well plates containing 50 µL of three-fold serial dilutions of Abs. An anti-CD47 mAb, 5F9-hIgG4, was used as a positive control for the assay. After 1 h incubation at room temperature (RT) without agitation, plates were put on a mirror and hemagglutination assessed visually. Samples with a pellet were considered negative for hemagglutination and those without a pellet were considered positive. Four donors were analyzed in two experiments.

Platelet activation

The ability of NILK-2401 to induce platelet activation in whole blood from ten human healthy donors (Hôpitaux Universitaires de Genève) was measured using the upregulation of the surface marker CD62P as readout. Briefly, 5 µL of whole blood was incubated with 10 µL of each sample (prepared at 2x) for 15 min at RT. Abs were added at different concentrations (0, 0.02, 0.2, 2, 20 and 200 µg/mL). Adenosine diphosphate (Sigma-Aldrich) at 10 µM and 1 µM as well as anti-CD9 at a concentration of 10 µg/mL (ALB6; Santa Cruz Biotechnology) were used as positive controls. Then, 10 µL of anti-CD41a-PE (BD Biosciences) and 10 µL of anti-CD62P-APC (BD Biosciences) were added and incubated for 15 min. at RT. Finally, 500 µL of CellFix (BD Biosciences, diluted 1/10 in water) were added and 200 µL of each sample was transferred in a U-bottom 96-well plate suitable for CytoFLEX acquisition. Platelets were identified by CD41a-PE positive staining. Platelet activation was assessed by the percentage of CD62P-positive platelets.

Cytokine release

In vitro cytokine release was assessed using whole blood from healthy human donors (Hôpitaux Universitaires de Genève) with minimal dilution by the test Abs (95% v/v blood) in aqueous presentation. Briefly, 10 µL of NILK-2401 and control Abs (i.e., anti-EGFR mAb Cetuximab [Farma Mondo] and CEACAM5xCD3 BsAb MEDI-565 [Absolute Antibodies]) at 50 µg/mL were incubated with 190 µL of human whole blood from 12 individuals. PBS was used as background control. Plates were incubated for 24 h at 37°C. Afterwards, plates were centrifuged, and plasma was harvested. Plasma samples were kept at -80°C until cytokine measurement. Interferon-γ (IFNγ), tumor necrosis factor α (TNFα), and interleukin 6 (IL-6) levels were measured with the U-PLEX® Development Packs (18151-v8-2020Mar) MSD kit, following supplier recommendations.

Pharmacokinetic (PK) and tolerability studies

Cynomolgus monkeys. A single-dose study with 6-week observational period was conducted in cynomolgus monkeys (*Macaca fascicularis*) to evaluate the *in vivo* tolerability and pharmacokinetic profile of NILK-2401 when injected IV (Accelera S.r.l., Italy). NILK-2401 was administered to male and female cynomolgus monkeys (n=2/sex/group) at doses of 0.5 or 20 mg/kg. Animals were observed for six weeks regarding changes in

clinical signs (including local tolerance), body weights, food intake, safety pharmacology (neurobehavioral observations, ECGs, blood pressure and respiratory rate), clinical pathology (hematology, coagulation, and clinical chemistry) and PK. Post-mortem examinations, including necropsy, organ weights, and collection of selected organs/tissues for possible histological examination, were performed on Day 43 at the end of the 6-week observation period.

Samples to assess the PK of NILK-2401 linked to anti-CD47 and Fc-binding were collected at pre-dose and at 0.25, 1, 4, 8, 24, 48, 72, 120, 168, 336, 504, 672, 840, and 1,008 hours after dosing.

Human FcRn Tg32. The study was performed in accordance with the Swiss Veterinary Office guidelines and approved by the Cantonal Veterinary Office (Geneva, Switzerland; #GE36). B6.Cg-Fcgrt^{tm1Dcr}Tg(FCGRT)32Dcr/DcrJ male mice (The Jackson Laboratory) of 7-8 weeks of age were injected with NILK-2401 IV at dose of 0.5 or 20 mg/kg (n=12 each) once in the tail vein. Mice were controlled daily for clinical symptoms and potential adverse events. Furthermore, mice were weighed at 0, 24, 48, 96, 168, 240, 336, 504, and 672 hours post-dose. Percentage of body weight was calculated compared to body weight at T=0 (100%).

PK samples were collected at 0.25, 6, 24, 48, 96, 168, 240, 336, 504, and 672 hours post-dose, from three animals/timepoint, in order to generate composite PK profiles.

PK analysis

Quantification of NILK-2401 in serum samples was performed at LCB using a generic pharmacokinetic assay based on MSD. Raw data and corresponding concentrations were generated with MSD Sector S600 instrument using MSD WorkBench 4.0 software for acquisition and analysis.

Non-compartmental analysis (NCA) was performed at Calvagone Sarl (France) using SAS software on mean concentration data, calculated for each timepoint and dose group using all values, and if applicable, repeated excluding outliers.

Immunogenicity assessment

The testing for potential immunogenicity of NILK-2401 was performed at the Département médicaments et technologies pour la santé, Université Paris-Saclay, CEA, INRAE, France. Overall, a panel of 16 healthy donors with different HLA-DR allotypes as assessed by HLA typing were used in the CD4 T-cell immunogenicity assay, among which only the 14 donors responded to Keyhole limpet hemocyanin (KLH) were retained for use in the data analysis for NILK-2401.

Buffy-coats from healthy individuals were provided by the Établissement Français du Sang (EFS, Rungis, France) after informed consent following EFS guidelines. PBMCs were isolated by Ficoll-Paque PLUS density gradient centrifugation (GE healthcare). The HLA-DR genotypes were determined by next generation sequencing (DKMS Life Science Lab GmbH, Germany) after DNA extraction from PBMCs with Nucleospin Blood Quick Pure (Macherey Nagel). Monocyte-derived immature dendritic cells (iDCs) were generated from Percoll gradient of PBMCs after 4- or 5-day culture in AIM-V medium (Invitrogen) supplemented with 1,000 units/mL rhIL-4 and rhGM-CSF (both from R&D Systems). iDCs were loaded with either NILK-2401 BsAb, Adalimumab or Trastuzumab mAbs or KLH at a concentration of 1 µM diluted in AIM-V medium supplemented with 1 µg/mL LPS and 10 µg/mL R848 and incubated overnight at 37°C. CD4 T-cells were isolated from autologous non-adherent PBMCs by positive selection using magnetic labeling with anti-CD4 mAbs conjugated to magnetic microbeads followed by magnetic cell sorting, as recommended by the manufacturer (Miltenyi Biotec). Mab-loaded DCs were washed twice with 100 µL AIM-V medium and then with 100 µL IMDM (Invitrogen) supplemented with 10% human AB serum (Sigma), 0.24 mM glutamine, 0.55 mM asparagine, 1.5 mM arginine (all amino acids from Sigma-Aldrich), 50 U/mL penicillin, and 50 µg/mL streptomycin (Invitrogen) (complete IMDM). 2×10^5 autologous CD4 T-cells were added to the round bottom wells of culture plates in 200 µL complete IMDM containing 1,000 U/mL IL-6 and 10 ng/mL IL-12 (both R&D systems). Twenty-four wells were seeded for each antibody and eight wells for KLH. CD4 T-lymphocytes were re-stimulated at days 7 and 14 with $1\text{-}3\times10^4$ autologous DCs freshly loaded with the appropriate protein and grown in complete IMDM medium supplemented with 10 U/mL IL-2 (R&D Systems) and 5 ng/mL IL-7 (R&D

Systems). The specificity of the CD4 T-cell lines was assessed at least 5 days after the last stimulation by IFNγ ELISPOT.

Multiscreen hemagglutinin (HA) 96-well plates (Merck Millipore) were coated overnight at 4°C with 2.5 µg/mL anti-human IFNγ mAb (1-D1K; Mabtech) in PBS (Invitrogen). Wells were saturated two hours at 37°C with complete IMDM and washed with PBS. Antibodies were loaded at a concentration of 3 µM onto autologous iDCs in AIM-V supplemented with 1.7 U/mL IL-7 for 4h at 37°C. KLH were used at a concentration of 1 µM, only. iDCs (5x103/well) were used as antigen-presenting cells and co-cultured in the plates with approximately $5x10^4$ CD4 T-cells in AIM-V supplemented with 1.7 U/mL IL-7. After overnight incubation at 37°C and washings, plates were sequentially treated with 0.25 µg/mL biotinylated anti-human IFNγ mAb (7-B6-1; Mabtech) in PBS/BSA 1%, Extravidin-phosphatase (dilution 1:3,000 in PBS/Tween-20 0.05%/BSA 1% (Sigma-Aldrich) and NBT/BCIP (Sigma-Aldrich). Spot number was determined by the AID ELISPOT Reader System (AID).

CD4+ T-cell lines were considered as specific when a spot count was 2-fold higher in the presence of the protein than in their absence, with a minimal difference of 25 spots. CD4+ T-cell precursor frequencies were estimated using the Poisson distribution according to the following formula:

Frequency = -Ln ((Number of non-specific CD4+ T-cell lines/Total number of CD4+ T-cell lines seeded))/(Number of CD4+ T-cells/well).

Combination assay

NILK-2401 plus NILK-2301, a CEACAM5-targeted T-cell BsAb (LamKap Bio alpha) (Seckinger et al., 2023), combination activity was tested based on a flow cytometric readout. In brief, human PBMCs from healthy donors were isolated from buffy coats (Hôpitaux Universitaires de Genève). An aliquot of these PBMCs was kept frozen in medium containing 90% FCS and 10% of cryoprotectant agent, to be used later as a source of T-cells. Another aliquot of the PBMCs was used to prepare monocyte-derived macrophages. After six days of differentiation, macrophages were plated in clear flat-bottom 96-well-plates and incubated at 37°C. Two days after plating, the autologous frozen PBMCs were thawed and added to the macrophages.

Target tumor cells (MKN-45 and LS174T) were stained with CellTrace Violet (ThermoFisher Scientific) and opsonized with the combination of NILK-2301 and NILK-2401 composed of a dose range of NILK-2301 and different fixed doses of NILK-2401. Opsonized target cells were added to the plate containing macrophages and the corresponding autologous PBMCs. Plates were incubated for 48 hours or 72 hours, depending on the cells line, at 37°C.

After incubation, floating cells in supernatant were collected and adherent cells were detached with trypsin (Sigma-Aldrich) and recovered with centrifugation. Floating and attached cells from the same well were pooled and stained with a labeled anti-CD14-APC antibody (ThermoFisher Scientific) to identify the macrophages during analysis. At the end of the staining, Sytox green (ThermoFisher Scientific), a viability marker, was added to eliminate dead cells from analysis. Cells were analyzed using flow cytometry (CytoFLEX; Beckman Coulter) and acquisitions were normalized to analyze the same sample size for each assay condition. The number of live target cells was calculated as follows:

Live target cells = total CellTrace-positive live cells - CD14/CellTrace double positive live cells.

SUPPLEMENTARY FIGURES

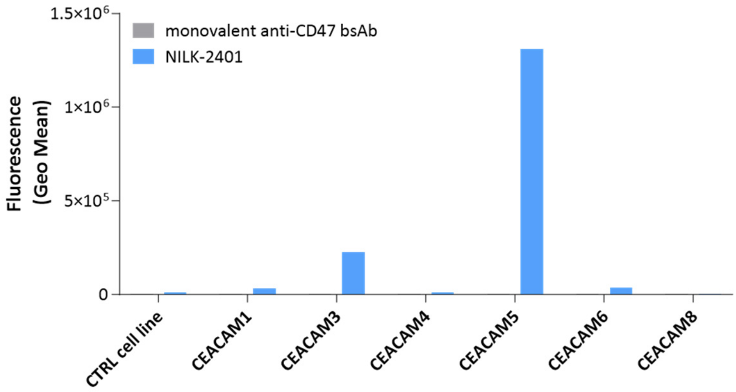

Supplementary Figure S1. Binding to different CEACAMs transiently expressed on PEAK cells. NILK-2401 showed a weak cross-reactivity to human CEACAM3 using PEAK cells transfected with different members of the CEACAM protein family by flow cytometric binding assay. See also Supplementary Table S6. Non-transfected PEAK cells were used as negative control (CTRL). BsAb, bispecific antibody.

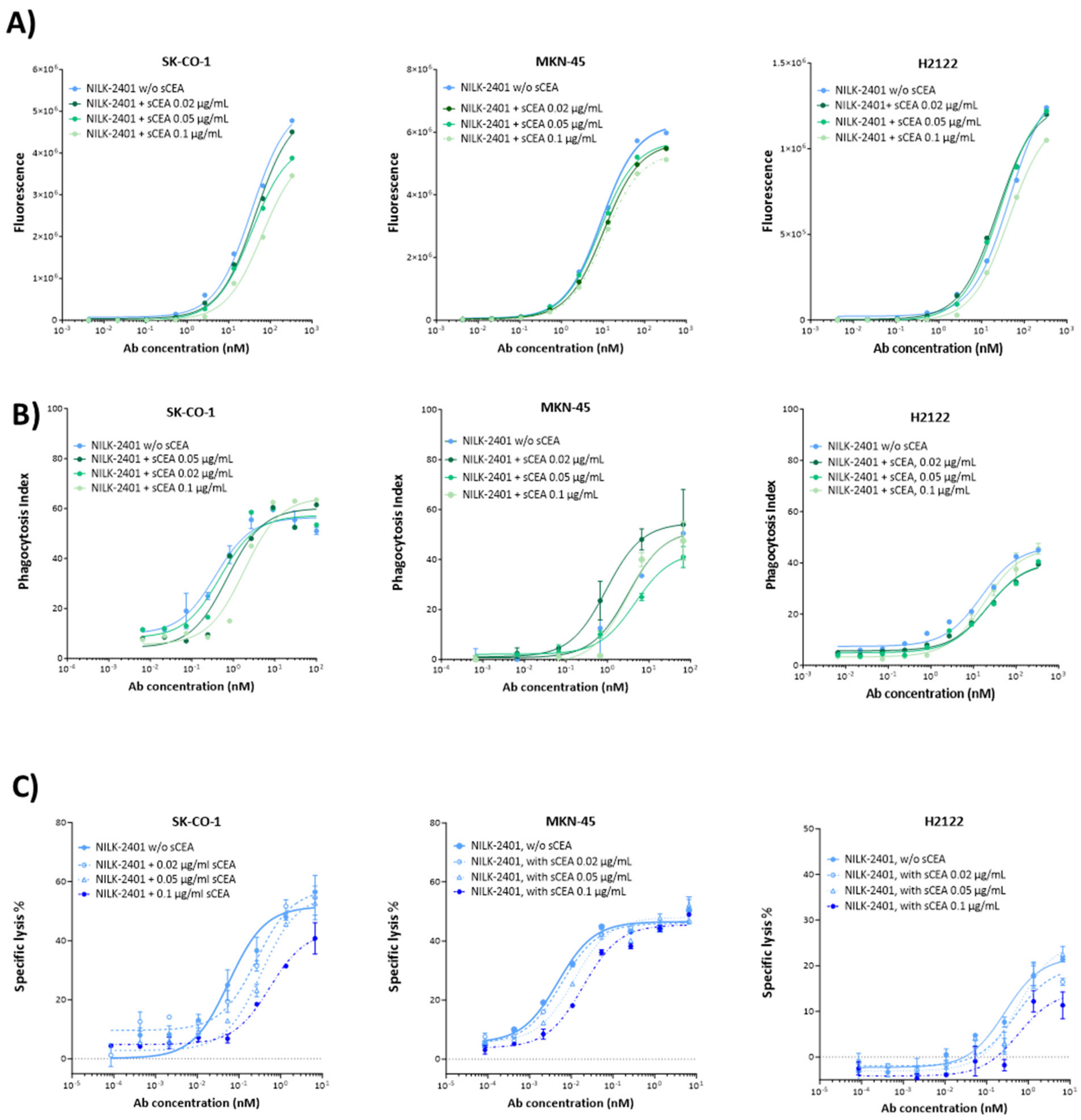

Supplementary Figure S2. Impact of sCEACAM5. A) Binding of NILK-2401 to SK-CO-1, MKN-45, and H2122 cells, as well as B) corresponding ADCP (exemplary data of one donor of at least three donors), and C) ADDC activity (exemplary data of one donor of at least three donors). Data are represented as mean ± standard deviation. sCEA, soluble CEACAM5. Ab, antibody.

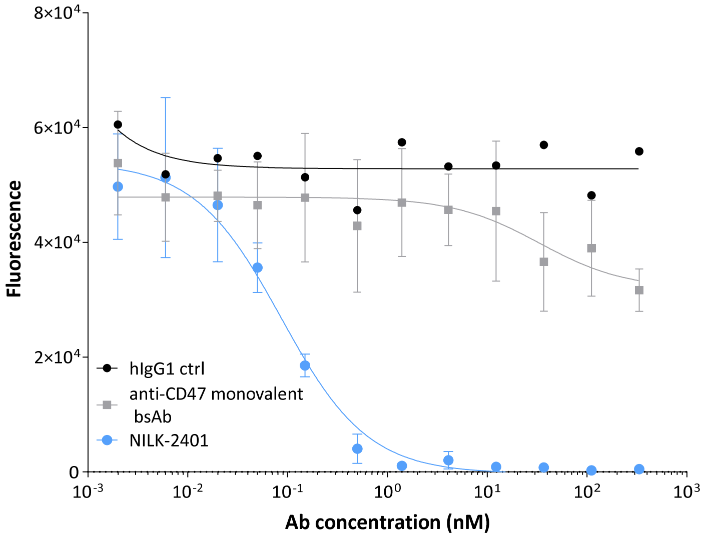

Supplementary Figure S3. CD47/SIRPα blocking activity of NILK-2401. NILK-2401 inhibited the interaction of the human SIRPα-Fc with CD47 expressed on the cell surface of MKN-45 target cells in a dose-dependent manner (blue line), with a higher blocking activity than the corresponding CD47 monovalent control (ctrl; grey line), indicating that efficient CD47 inhibition is dependent on CEACAM5 co-engagement. Exemplary data of two independent experiments. Data represented are mean ± standard deviation. BsAb, bispecific antibody.

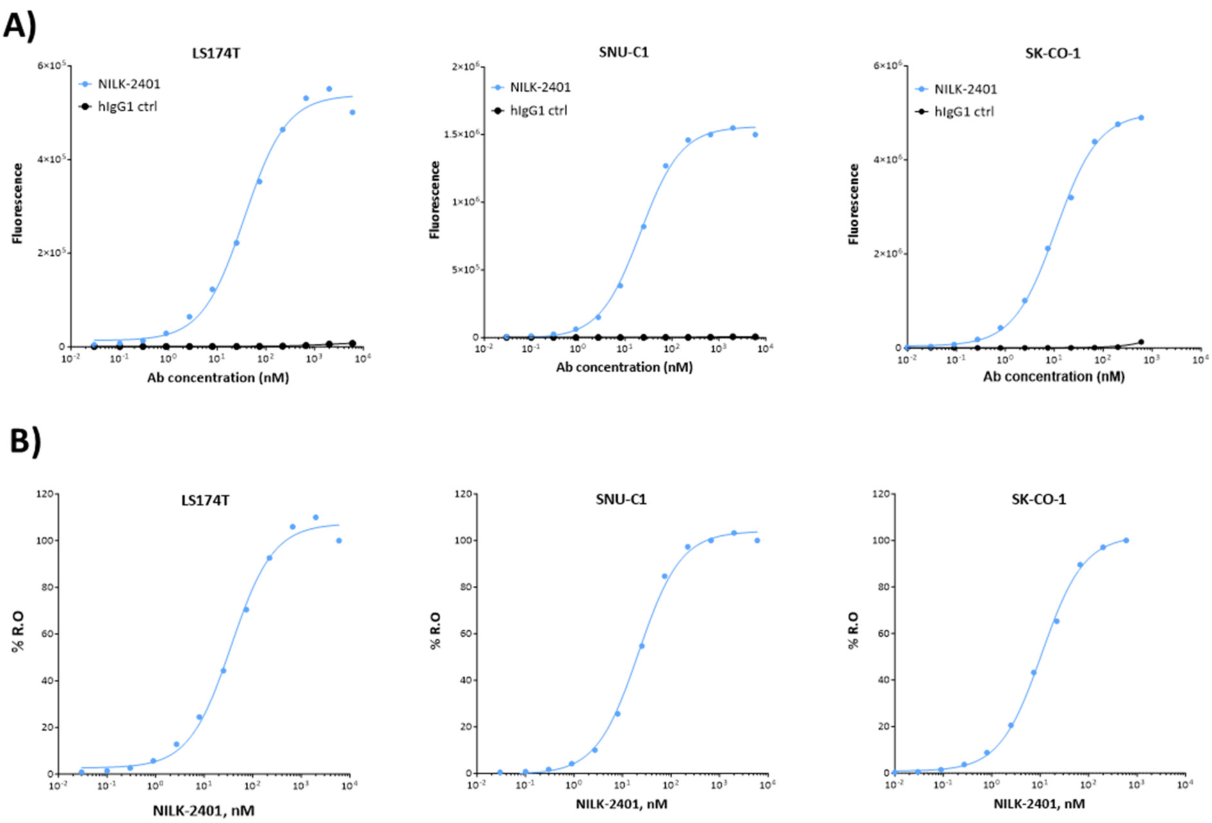

Supplementary Figure S4. Binding of NILK-2401 to CEACAM5-positive cell lines and corresponding receptor occupancy. A) Binding was assessed for colorectal cancer cell lines with different CEACAM5-expression levels, i.e., LS174T, SNU-C1, and SK-CO-1 by using flow cytometry. B) Corresponding percentage of receptor occupancy of NILK-2401 was measured for the same cell lines. An inverse correlation between the level of CEACAM5 expression on the target cells and the RO_{50} of NILK-2401 to the target cells was observed. One experiment was performed for each cell line.

Supplementary Figure S5

A)

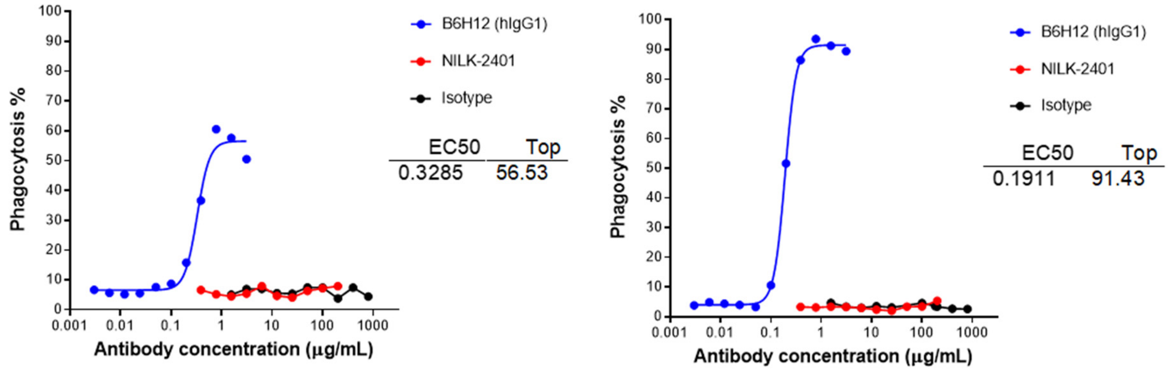

B)

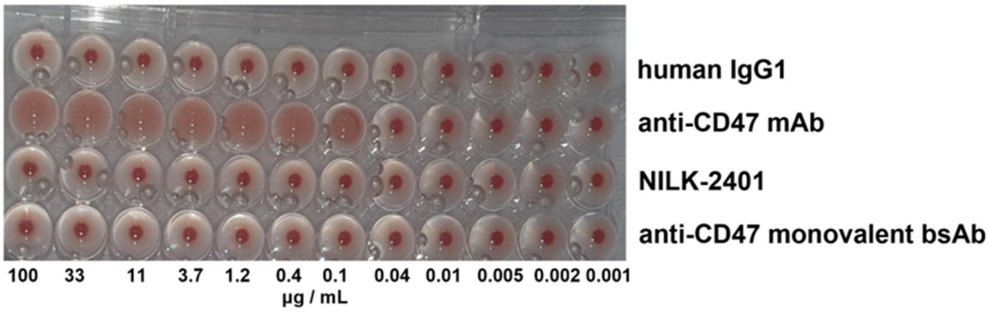

C)

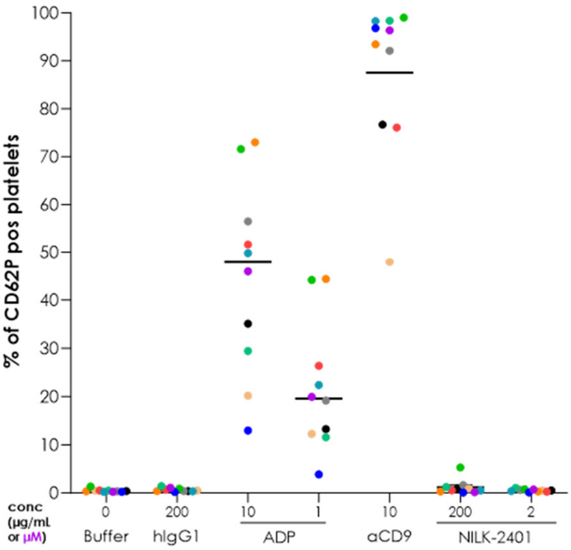

Supplementary Figure S5. Erythrophagocytosis, hemagglutination, and platelet aggregation. A) Concentration-response curves of erythrophagocytosis induced by NILK-2401 and B6H12 antibodies vs. isotype control in the presence of IgG excess. Data for two different donors are shown. EC_{50} and top values are indicated for the positive control (hB6H12). B) Macroscopic assessment of human hemagglutination induced by CD47-targeting antibodies, i.e., 5F9-hIgG4 and NILK-2401. A human IgG1 antibody was used as negative control. Each condition is tested in simplicate and blood samples from four healthy volunteer donors were tested. Exemplary data for one of four donors are shown. C) Platelet activation was assessed on ten different donors using the expression of CD62P as a readout. Dots with the same color come from the same donor. Induction of CD62P expression was only observed when using the positive controls, i.e., following incubation with adenosine diphosphate (ADP; tested at 10 or 1 μM) or anti-CD9, but not by NILK-2401. hIgG1, human immunoglobulin G1.

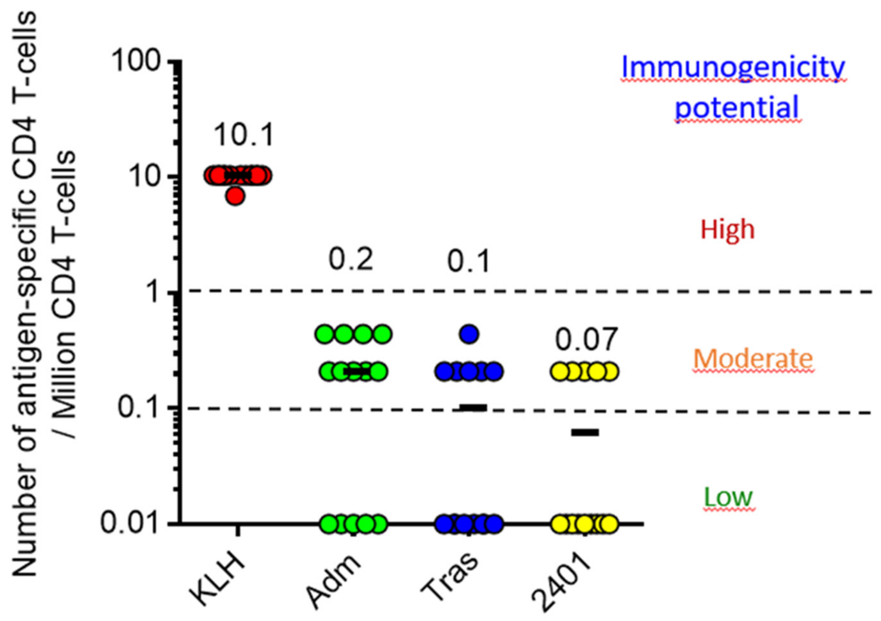

Supplementary Figure S6. Size of the pre-existing protein-specific CD4 T-cell repertoire in the immunogenicity assessment. Estimate of the number of pre-existing protein-specific CD4 T-cells was done by considering the Poisson's distribution of the cells at the initiation of the culture. KLH, Keyhole limpet haemocyanin; Adm, Adalimumab (anti-TNFα monoclonal antibody); Tras, Trastuzumab (anti-HER2 monoclonal antibody); 2401, NILK-2401 bispecific antibody.

SUPPLEMENTARY TABLES

Supplementary Table S1. Quantification of CEACAM5 and CD47 surface expression. Cell lines and normal epithelial cells used with their tissue origin and number of surface CEACAM5 and CD47 molecules/cell as assessed by QIFIKIT® assay.

Cell line	Tissue origin	CEACAM5/cell	CD47/cell
**SK-CO-1**	Colorectal	257,000	105,000
**SNU-C1**	Colorectal	85,000	68,000
**H508**	Colorectal	80,000	81,000
**LS174T**	Colorectal	26,000	57,000
**MKN-45**	Gastric	155,000	135,000
**SNU-16**	Gastric	110,000	27,000
**H727**	Lung	114,000	29,000
**H2122**	Lung	33,000	35,000
**CCD841CoN***	Colon	<LD	71,000
**HBEpiC***	Lung	<LD	46,000
**A549**	Lung	<LD	48,000
**MC38-hCD47-hCEA#**	Colon	203,000	40,000

*Isolated from non-cancerous human tissue. #Mus musculus. LD, limit of detection.

Supplementary Table S2. EC_{50} and maximal plateau from ADCP and ADCC assays [%] with CEACAM5-positive tumor cell lines.

	NILK-2401			
	ADCP		ADCC	
	EC_{50} nM	Max plateau	EC_{50} nM	Max plateau
**SK-CO-1**	1.05 ± 0.37	42.01 ± 12.78	0.1 ± 0.05	31.8 ± 15.7
**SNU-C1**	1.23 ± 0.7	22.24 ± 4.27	0.13 ± 0.06	24.9 ± 13.5
**LS174T**	12.51 ± 5.65	43.79 ± 18.83	0.04 ± 0.02	16.8 ± 10.7
**MKN-45**	12.93 ± 6.92	56.68 ± 9.93	0.07 ± 0.06	35.7 ± 10.6
**SNU-16**	0.38 ± 0.24	21.99 ± 3.71	0.25 ± 0.13	20.5 ± 7.5
**H727**	1.02 ± 0.35	35.87 ± 7.8	0.15 ± 0.07	8.7 ± 4.6
**H2122**	25.84 ± 15.06	28.87 ± 16	0.09 ± 0.05	27.3 ± 16.6
**H508**	ND	ND	0.08 ± 0.03	19 ± 7.1

ND, not done.

Supplementary Table S3. MFI values regarding NILK-2401 binding to various cell populations from human and cynomolgus whole blood. Shown are average values ± standard deviation for NILK-2401, CD47 monovalent, or IgG1 isotype control binding to human (n=2) and cynomolgus monkey (n=4; two males and two females) whole blood samples.

	Ab [3 µg/mL)	Neutrophils	Monocytes	B-cells	T-cells	NK cells	Platelets
Human	**NILK-2401**	2,486 ± 78	1,162 ± 59	636 ± 115	789 ± 7	1,205 ± 372	345 ± 93
	**CD47 monovalent**	1,574 ± 48	1,072 ± 188	619 ± 33	740 ± 72	1,094 ± 404	216 ± 48
	**IgG1 isotype control**	1,235 ± 17	922 ± 82	454 ± 138	482 ± 42	465 ± 67	71 ± 15
	Ab [10 µg/mL)	Neutrophils	Monocytes	B-cells	T-cells	NK cells	Platelets
	**NILK-2401**	5,452 ± 23	1,894 ± 43	1,068 ± 139	1,445 ± 75	2,700 ± 899	828 ± 223
	**CD47 monovalent**	2,627 ± 18	1,492 ± 208	1,095 ± 30	1,339 ± 95	2,486 ± 934	486 ± 146
	**IgG1 isotype control**	1,606 ± 96	1,070 ± 98	444 ± 90	510 ± 44	489 ± 78	90 ± 7
	Ab [30 µg/mL)	Neutrophils	Monocytes	B-cells	T-cells	NK cells	Platelets
	**NILK-2401**	12,657 ± 407	3,876 ± 75	2,085 ± 21	3,118 ± 182	6,219 ± 1866	1,930 ± 428
	**CD47 monovalent**	5,661 ± 59	2,886 ± 185	2,213 ± 6.4	2,861 ± 142	5,802 ± 2127	1,093 ± 265
	**IgG1 isotype control**	2,539 ± 549	1,510 ± 175	471 ± 57	578 ± 83	606 ± 129	171 ± 20
Cynomolgus	Ab [3 µg/mL)	Neutrophils	Monocytes	B-cells	T-cells	NK cells	Platelets
	**NILK-2401**	1,091 ± 116	1,612 ± 304	648 ± 127	823 ± 223	1,050 ± 245	137 ± 30
	**CD47 monovalent**	1,109 ± 107	1,585 ± 314	661 ± 130	566 ± 95	936 ± 185	104 ± 25
	**IgG1 isotype control**	969 ± 71	1,324 ± 232	498 ± 59	399 ± 29	609 ± 51	51 ± 10
	Ab [10 µg/mL)	Neutrophils	Monocytes	B-cells	T-cells	NK cells	Platelets
	**NILK-2401**	1,733 ± 290	3,133 ± 580	1,199 ± 242	2,120 ± 834	2,564 ± 803	542 ± 53
	**CD47 monovalent**	1,568 ± 209	2,674 ± 352	1,193 ± 305	1,133 ± 279	2,120 ± 533	341 ± 38
	**IgG1 isotype control**	1,117 ± 129	1,628 ± 299	539 ± 43	464 ± 23	752 ± 42	105 ± 15
	Ab [30 µg/mL)	Neutrophils	Monocytes	B-cells	T-cells	NK cells	Platelets
	**NILK-2401**	3461 ± 586	6,362 ± 632	2,617 ± 422	3,749 ± 1473	5,669 ± 1891	1,329 ± 122
	**CD47 monovalent**	2754 ± 535	5,151 ± 574	2,352 ± 573	2,523 ± 946	4,772 ± 1453	904 ± 63
	**IgG1 isotype control**	1382 ± 238	2,362 ± 428	599 ± 67	546 ± 77	998 ± 121	226 ± 34

Supplementary Table S4. PK parameters for NILK-2401 after IV administration in cynomolgus monkeys. Data are shown for 0.5 mg/kg and 20 mg/kg.

	Animal ID	C_0	C_{max}	t_{max}	C_{last}	t_{last}	$AUC_{0\text{-}last}$	$AUC_{0\text{-}inf}$	λ_z	$t_{1/2z}$	CL	V_z
**0.5 mg/kg**		[µg/mL]	[µg/mL]	[h]	[µg/mL]	[h]	[µg.h/mL]	[µg.h/mL]	[1/d]	[d]	[mL/h/kg]	[mL/kg]
	1	9.66	9.07	0.25	0.039	1008	666	675	0.1018	6.81	0.741	175
	2	16.8	16.3	0.25	0.048	1008	1161	1174	0.0942	7.36	0.426	109
	3	12.7	12.4	0.25	0.029	1008	1038	1044	0.1125	6.16	0.479	102
**20 mg/kg**	Animal ID	C_0	C_{max}	t_{max}	C_{last}	t_{last}	$AUC_{0\text{-}last}$	$AUC_{0\text{-}inf}$	λ_z	$t_{1/2z}$	CL	V_z
		[µg/mL]	[µg/mL]	[h]	[µg/mL]	[h]	[µg.h/mL]	[µg.h/mL]	[1/d]	[d]	[mL/h/kg]	[mL/kg]
	5	1,055	1,560	8	0.083	504	47,996	48,000	0.4577	1.51	0.417	21.8
	6	1,368	1,618	4	1.4	1,008	89,762	89,993	0.1457	4.76	0.222	36.6
	7	1,140	1,102	0.25	0.062	504	47,036	47,039	0.4168	1.66	0.425	24.5
	8	489	473	0.25	0.58	1,008	27,024	27,263*	0.0582*	11.9*	0.734*	303*

* One condition on PK parameters was not fulfilled.

Supplementary Table S5. PK parameters for NILK-2401 after IV administration in Tg32 hFcRn mice.

Data are shown for 0.5 mg/kg and 20 mg/kg using all values as well as for 20 mg/kg without outliers.

Dose	C_0	C_{max}	t_{max}	AUC_{last}	AUC_{inf}	AUC_{extr}	λ_z	$t_{1/2z}$	CL
	[µg/mL]	[µg/mL]	[h]	[µg.h/mL]	[µg.h/mL]	[%]	[1/d]	[d]	[mL/h/kg]
0.5 mg/kg (all values)	13.7	13.3	0.25	1,438	1,765*	18.5	0.0603*	11.5*	0.283*
20 mg/kg (all values)	502	492	0.25	40,049	49,296*	18.8	0.0451*	15.4*	0.203*
20 mg/kg (without outliers)	502	492	0.25	45,921	54,748*	16.1	0.0607*	11.4*	0.183*

* At least one condition on PK parameters was not fulfilled.

Supplementary Table S6. Reference of commercial antibodies used as positive control to verify expression on PEAK cells. The expression level of each CEACAM on PEAK cells was assessed before the binding experiment by using flow cytometry. See also Supplementary Figure S1.

Target	Reference	Supplier
**CEACAM1**	FAB2244P	Bio-Techne
**CEACAM3**	LS-C485352	LSBio
**CEACAM4**	FAB7845R	Bio-Techne
**CEACAM5**	FAB41281P	Bio-Techne
**CEACAM6**	551478	Becton Dickinson
**CEACAM8**	561650	Becton Dickinson

REFERENCES

Liu, J., Wang, L., Zhao, F., Tseng, S., Narayanan, C., Shura, L., et al. (2015) Pre-Clinical Development of a Humanized Anti-CD47 Antibody with Anti-Cancer Therapeutic Potential. *PLoS One*, 10(9), e0137345.

Seckinger, A., Delgado, J. A., Moser, S., Moreno, L., Neuber, B., Grab, A., et al. (2017) Target Expression, Generation, Preclinical Activity, and Pharmacokinetics of the BCMA-T Cell Bispecific Antibody EM801 for Multiple Myeloma Treatment. *Cancer Cell*, 31(3), 396-410.

Seckinger, A., Majocchi, S., Moine, V., Nouveau, L., Ngoc, H., Daubeuf, B., et al. (2023) Development and characterization of NILK-2301, a novel CEACAM5xCD3 κλ bispecific antibody for immunotherapy of CEACAM5-expressing cancers. *Journal of Hematology & Oncology*, 16(1), 117.
